# RNAseqCovarImpute: a multiple imputation procedure that outperforms complete case and single imputation differential expression analysis

**DOI:** 10.1186/s13059-024-03376-7

**Published:** 2024-09-03

**Authors:** Brennan H. Baker, Sheela Sathyanarayana, Adam A. Szpiro, James W. MacDonald, Alison G. Paquette

**Affiliations:** 1 Department of Environmental and Occupational Health Sciences, University of Washington, Seattle, WA USA; 2 Center for Child Health, Behavior, and Development, Seattle Children’s Research Institute, Seattle, WA USA; 3 Department of Pediatrics, University of Washington, Seattle, WA USA; 4 Department of Epidemiology, University of Washington, Seattle, WA USA; 5 Department of Biostatistics, University of Washington, Seattle, WA USA; 6 Center for Developmental Biology and Regenerative Medicine, Seattle Children’s Research Institute, Seattle, WA USA

**Keywords:** Differential expression analysis, RNA-sequencing, Gene expression, Multiple imputation, Missing data

## Abstract

Missing covariate data is a common problem that has not been addressed in observational studies of gene expression. Here, we present a multiple imputation method that accommodates high dimensional gene expression data by incorporating principal component analysis of the transcriptome into the multiple imputation prediction models to avoid bias. Simulation studies using three datasets show that this method outperforms complete case and single imputation analyses at uncovering true positive differentially expressed genes, limiting false discovery rates, and minimizing bias. This method is easily implemented via an R Bioconductor package, RNAseqCovarImpute that integrates with the limma-voom pipeline for differential expression analysis.

**Supplementary Information:**

The online version contains supplementary material available at 10.1186/s13059-024-03376-7.

## Background

Missing data is a common problem in observational studies, as modeling techniques such as linear regression cannot be fit to data with missing points. Missing data is frequently handled using complete case (CC) analyses in which any individuals with missing data are dropped from the study. Dropping participants can reduce statistical power and, in some cases, result in biased model estimates. A common technique to address these problems is to replace or “impute” missing data points with substituted values. Typically, for a given covariate, missing data points are imputed using a prediction model including other relevant covariates as independent variables. In single imputation (SI), a missing value is replaced with the most likely value based on the predictive model. Statistical efficiency can be improved by including the outcome in the predictive model in addition to covariates. However, in this setting, SI methods can result in biased coefficients and over-confident standard errors [1]. Multiple imputation (MI) addresses this problem by generating several predictions, thereby allowing for uncertainty about the imputed data to propagate through the analysis. In a typical MI procedure: (1) *m* imputed data sets are created, (2) each data set is analyzed separately (e.g., using linear regression), and (3) estimates and standard errors across the *m* analyses are pooled using Rubin’s rules [2, 3].

To date, there has been no concerted effort to determine the most advantageous method for handling missing covariate data in transcriptomic studies. A large proportion of RNA-sequencing studies are conducted in in vitro or in vivo models and do not suffer from missing covariate data. Complete datasets are common in experimental studies with controlled conditions and a limited number of covariates. In an experimental setting, studies may employ two-group analyses with no additional variables or utilize covariates for which collecting data is trivial (e.g., sequencing batch and sex). However, the cost of sequencing has decreased over time [4], and transcriptomic data are already becoming more common in large human observational studies where missing data is a prevailing concern [5, 6]. Therefore, guidelines for handling missing data in this context are critically needed to facilitate the integration of transcriptomic and epidemiologic approaches.

While SI methods must omit the outcome from the imputation predictive model to avoid bias, the opposite is true of MI [7]. However, including the outcome in the MI predictive model can be problematic in “omics” studies with high dimensional data. Fitting an imputation model where the number of independent variables is far greater than the number of individuals in the study is generally not feasible. For instance, in RNA-sequencing studies with tens of thousands of genes, an equal or greater number of participants may be needed to apply a standard MI procedure.

To ensure that outcome data are included in the predictive model (a requirement of MI to avoid bias [7]), one solution is to make one set of *m* imputed datasets per gene, where expression data for a single gene is included in the predictive model. Then, each set of imputed data can be used to estimate differential expression of the gene that was used in that set’s predictive modeling. However, the generation of tens of thousands of sets of imputed data is computationally intensive and may require an unfeasible amount of model checking and diagnostics. In epigenetic studies of DNA methylation at CpG cites, this approach has been modified to be less computationally intensive by using groups of CpG sites together to impute missing data [8, 9]. We propose an alternative solution for applying MI to high dimensional gene expression data, which is to utilize principal component analysis (PCA) to reduce the dimensionality of the transcriptome. Then, the top PCs can be included in the MI prediction model when imputing missing covariates, satisfying the requirement that outcome information is included in the MI predictive models.

Here, we developed the first method to our knowledge to make MI compatible with high dimensional transcriptomic data. We created an R package (RNAseqCovarImpute) that is fully compatible with the popular limma-voom [10–12] differential expression analysis pipeline. We conducted a simulation study to compare the performance of MI as implemented in RNAseqCovarImpute with random forest SI and CC analyses. Finally, we applied RNAseqCovarImpute to two analyses involving (1) the placental transcriptome associated with maternal age, and (2) the blood platelet transcriptome associated with colorectal carcinoma.

## Results

### Multiple imputation and differential expression analysis in the RNAseqCovarImpute package

The RNAseqCovarImpute package includes two methods accommodating the requirement of MI that the outcome data are included in the MI predictive models. The first method surmounts the problem of high-dimensional outcome data by binning genes into smaller groups to analyze pseudo-independently (MI Gene Bin method, see Additional file 1: Supplemental Methods). Analyzing smaller bins of genes independently lowers the dimensionality of the outcome gene expression data, allowing us to include it in the MI predictive modeling. However, binning genes into smaller groups is computationally inefficient, as it requires that the MI and limma-voom analysis is run many times (typically hundreds).

**Fig. 1:**
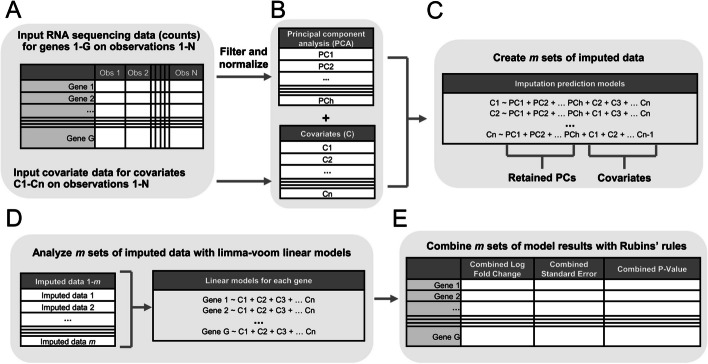
Overview of RNAseqCovarImpute multiple imputation differential expression analysis. **A** Inputs are covariates, including the predictor of interest and adjustment variables, and RNA-sequencing counts that are filtered to remove low counts and normalized as log-counts per million (logCPM). The logCPM calculation uses the effective library sizes calculated using the weighted trimmed mean of M-values method. **B** Principal component analysis (PCA) is used to reduce the dimensionality of the count matrix and Horn’s parallel analysis determines the number of PCs (1-h) to retain. Retained PCs (PC1-PCh) are added to the input dataset of covariates (C1-Cn). **C** Multiple imputation imputes missing covariate data *m* times (RNA-sequencing data are not imputed). All covariates and all retained PCs are included in the imputation prediction models. **D** Associations are estimated between the covariates and gene expression, according to the user’s statistical model design of interest, separately within each *m* imputed dataset using voom followed by lmFit followed by eBayes functions. In this example, the design is a multivariable linear model including all covariates C1-Cn. **E** Combine across *m* sets of model results using Rubin’s rules to produce combined log fold changes, standard errors, and *P* values for each term in the design

A second method uses PCA to avoid binning genes while still retaining outcome information in the MI models. The MI PCA method implements covariate MI in gene expression studies by (1) performing PCA on the normalized log-counts per million (logCPM) for all genes using the Bioconductor “PCAtools” package [13]; (2) creating *m* imputed datasets where the imputation predictor matrix includes all covariates and the optimum number of PCs to retain; (3) conducting the standard limma-voom differential expression analysis pipeline in R with the “limma::voom” followed by “limma::lmFit” followed by “limma::eBayes” functions [10–12] on each *m* imputed dataset; (4) pooling the results with Rubin’s rules to produce combined coefficients, standard errors, and *P* values; and (5) adjusting *P* values for multiplicity to account for false discovery rate (FDR) (Fig. 1; see “ Methods” for details). Various methods for determining the number of PCs to retain in the MI prediction model can be utilized. For example, Horn’s parallel analysis, which retains PCs with eigenvalues greater than eigenvalues of random data [14, 15], utilizing an 80% explained variation cutoff, or the elbow method.

Three versions of MI PCA using different criteria to determine the number of retained PCs were compared with the MI Gene Bin approach (“ Methods”). MI PCA using Horn’s parallel analysis performed better than the MI Gene Bin and other MI PCA methods. All methods had similar true positive rates (TPRs), while MI PCA horn had the lowest false positive rates (FPRs) across most scenarios (Additional file 1: Supplemental Results, Additional file 1: Figs. S2–S4). Among the methods for retaining PCs, results were relatively comparable when missing data were minimal. For instance, for the ECHO-PATHWAYS dataset, Horn’s parallel analysis retained 35 PCs, an 80% variance explained cutoff retained 213 PCs, and the elbow method retained 15 PCs. Despite substantial differences in the number of retained PCs, all methods had similarly high TPRs and good FPR control at approximately 0.05 when there was only 5–15% missing data (Additional file 1: Fig. S2). However, when levels of missing data were higher, FPRs were consistently controlled at 0.05 for Horn’s parallel analysis, but not for the 80% variance explained or elbow approaches (Additional file 1: Fig. S2). Thus, MI PCA using Horn’s parallel analysis was selected as the MI method of choice (hereinafter the “RNAseqCovarImpute” method).

### Performance on three real datasets following simulations of missing covariate data

Three large real-world RNA-sequencing datasets encompassing multiple tissue types and a diverse range of covariates were utilized to compare RNAseqCovarImpute, SI, and CC differential expression analysis (“ Methods”). 

The ECHO prenatal and early childhood pathways to health (ECHO-PATHWAYS) dataset (dbGaP phs003619.v1.p1 and phs003620.v1.p1) includes RNA-sequencing of placentas sampled at delivery from socioeconomically and racially/ethnically diverse participants from two regionally distinct birth cohorts in Washington (Seattle and Yakima) and Tennessee (Memphis), USA [5]. For the ECHO-PATHWAYS dataset (N = 994), maternal age served as the predictor of interest in differential expression analysis, while covariates included fetal sex, RNA-sequencing batch, maternal tobacco use during pregnancy, maternal alcohol use during pregnancy, and family income. 

The non-small cell lung cancer (NSCLC) dataset (EMBL-EBI: E-GEOD-81089) includes RNA-sequencing of both lung tumor and non-malignant tissues sampled from patients diagnosed with NSCLC being surgically treated from 2006 to 2010 at the Uppsala University Hospital, Sweden [16]. For the NSCLC dataset (N = 670), the predictor of interest was sex, while covariates included participant age, participant smoking status, and sampling site (tumor versus non-malignant). The non-small cell lung cancer (NSCLC) dataset (EMBL-EBI: E-GEOD-81089) includes RNA-sequencing of both lung tumor and non-malignant tissues sampled from patients diagnosed with NSCLC being surgically treated from 2006 to 2010 at the Uppsala University Hospital, Sweden [16]. For the NSCLC dataset (N = 670), the predictor of interest was sex, while covariates included participant age, participant smoking status, and sampling site (tumor versus non-malignant).

The Epstein-Barr virus (EBV) dataset (EMBL-EBI: E-MTAB-7805) analyzed primary cultures of human B lymphocytes obtained from adenoid tissue [17]. For the EBV dataset (*N* = 384), the predictor of interest was time elapsed in culture, while covariates included EBV infection status and individual donor source.

The ECHO-PATHWAYS dataset included 14,026 genes after filtering, of which 2517 were significantly associated with maternal age in the full data model (true positives) while adjusting for covariates. Following the full data model, simulations to induce missingness in the covariate data were performed 10 times per level of missing data and missingness mechanism (“ Methods”). Patterns of simulated missing data depended on the missingness mechanism. When data were simulated to be missing at random (MAR) or missing not at random (MNAR), the maternal alcohol and family income variables had strong influence over patterns of missing data as intended. For example, in the simulated datasets where 55% of individuals had at least one missing data point, the average rate of individuals with at least one missing data point was 91% among alcohol users but only 50% among those reporting no alcohol use. Family income also impacted missingness: the missing data rate was 37% among those in the bottom quartile of family income, but 75% among those in the top quartile. These patterns of missingness were identical between the MAR and MNAR mechanisms, the only difference being that SI and RNAseqCovarImpute had access to these variables (while imputing data) under MAR, while these variables were masked under MNAR. Thus, under MNAR, unobserved data influenced the patterns of missingness. When data were simulated to be missing completely at random (MCAR), missingness did not depend on alcohol use, family income, or any other covariate. For example, in the 10 simulated datasets where 55% of individuals had at least one missing data point, the rate of individuals with at least one missing data point was 55% among alcohol users, 53% among those reporting no alcohol use, 56% among those in the bottom quartile of family income, and 54% in the top quartile of family income.

**Fig. 2:**
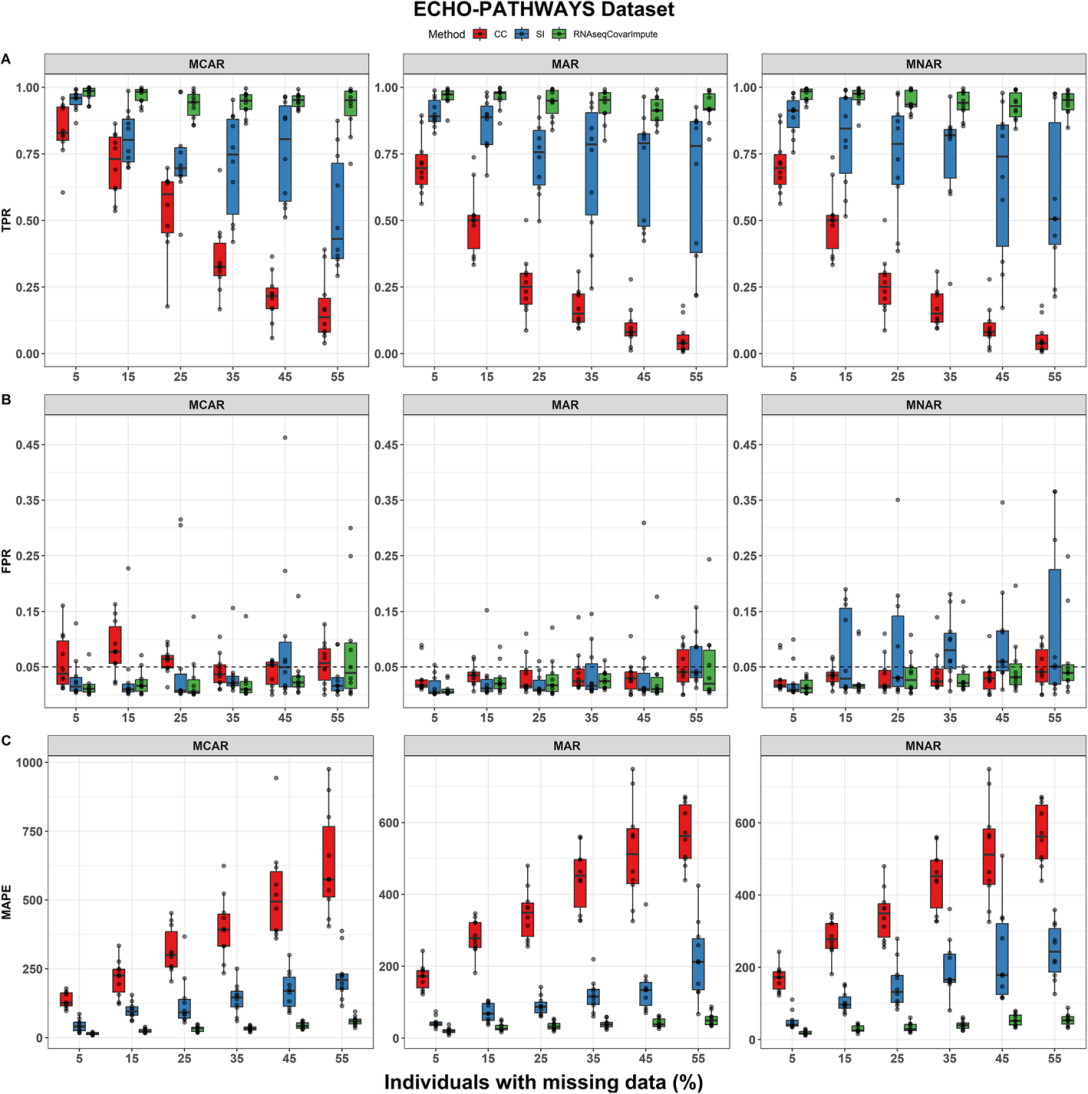
Performance of missing data methods on ECHO-PATHWAYS dataset. **A** True positive rate (TPR), **B** false positive rate (FPR), and **C** mean absolute percentage error (MAPE) shown for complete case (CC), single imputation (SI), and RNAseqCovarImpute multiple imputation differential expression analyses on ten datasets with simulated missingness per missingness mechanism per level of missingness. Box (median and interquartile range) and whiskers (1.5* interquartile range) shown along with one point per simulation. Dashed line at target FPR of 0.05

In differential expression analysis using the ECHO-PATHWAYS dataset, RNAseqCovarImpute was the best performer, with the highest TPR, lowest FPR, and lowest mean absolute percentage error (MAPE) across most scenarios, especially with increasing levels of missing data (Fig. 2). For example, when 55% of participants had at least one missing data point, the TPR ranged from 0.713 to 0.994 for RNAseqCovarImpute, from 0.214 to 0.977 for SI, and from 0.006 to 0.391 for CC (Fig. 2A). FPR was well-controlled under 0.05 in most scenarios, but more consistently so for RNAseqCovarImpute. For example, the median FPR was always < 0.05 for RNAseqCovarImpute, while there were some cases of high FPRs for the CC method when data were MCAR, and for the SI method when data were MNAR (Fig. 2B). MAPE was lower for RNAseqCovarImpute in almost every scenario (Fig. 2C).

**Fig. 3:**
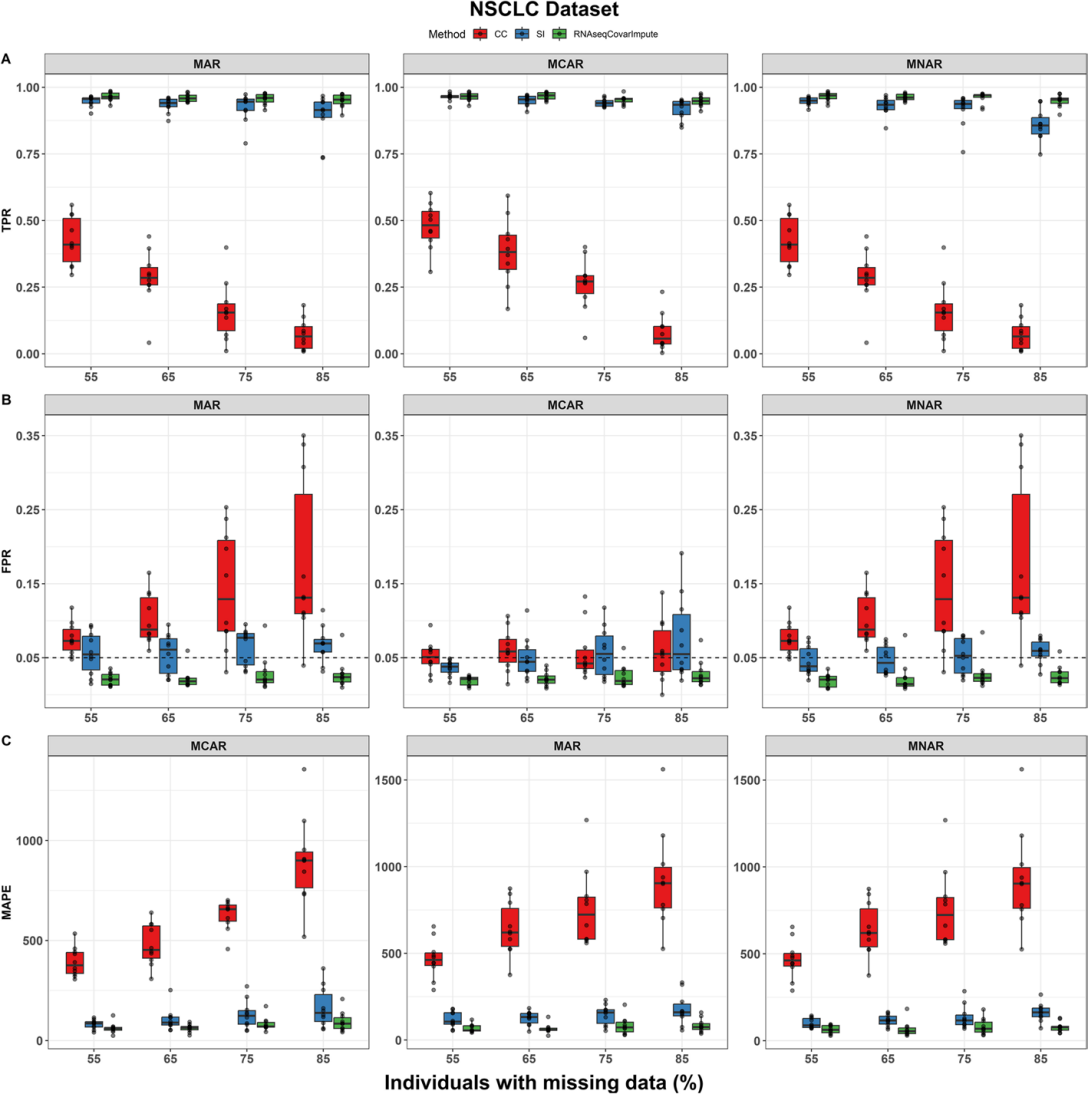
Performance of missing data methods on NSCLC dataset. **A** True positive rate (TPR), **B** false positive rate (FPR), and **C** mean absolute percentage error (MAPE) shown for complete case (CC), single imputation (SI), and RNAseqCovarImpute multiple imputation differential expression analyses on ten datasets with simulated missingness per missingness mechanism per level of missingness. Box (median and interquartile range) and whiskers (1.5* interquartile range) shown along with one point per simulation. Dashed line at target FPR of 0.05

The NSCLC dataset included 12,353 genes after filtering, of which 5718 were significantly associated with sex in the full data model (true positives) while adjusting for covariates. After inducing missingness in the covariate data, RNAseqCovarImpute was the best performer with the highest TPR, lowest FPR, and lowest MAPE across most scenarios, especially with increasing levels of missing data (Fig. 3). For example, when 85% of participants had at least one missing data point, the TPR ranged from 0.933 to 0.987 for RNAseqCovarImpute, from 0.902 to 0.984 for SI, and from 0.296 to 0.604 for CC (Fig. 3A). Many scenarios had FPR > 0.05 for CC, while FPR was well-controlled at approximately 0.05 for SI, and consistently below 0.05 for RNAseqCovarImpute (Fig. 3B). As with FPR, MAPE was lowest for RNAseqCovarImpute, followed by SI and CC, respectively (Fig. 3C).

**Fig. 4:**
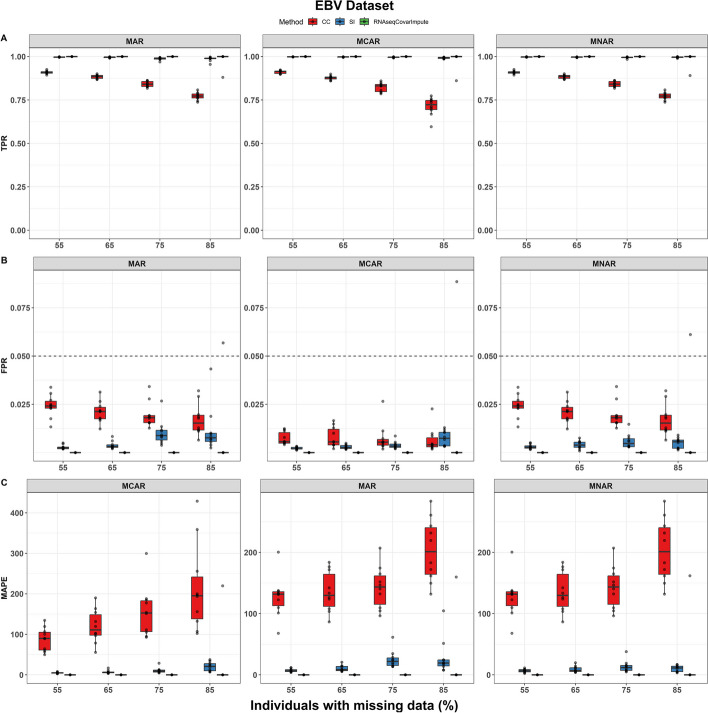
Performance of missing data methods on EBV dataset. **A** True positive rate (TPR), **B** false positive rate (FPR), and **C** mean absolute percentage error (MAPE) shown for complete case (CC), single imputation (SI), and RNAseqCovarImpute multiple imputation differential expression analyses on ten datasets with simulated missingness per missingness mechanism per level of missingness. Box (median and interquartile range) and whiskers (1.5* interquartile range) shown along with one point per simulation. Dashed line at target FPR of 0.05

The EBV dataset included 8677 genes after filtering, of which 7449 were significantly associated with time in the full data model (true positives) while adjusting for covariates. As with the datasets above, after inducing missingness in the covariate data, RNAseqCovarImpute was the best performer with the highest TPR, lowest FPR, and lowest MAPE across most scenarios (Fig. 4).

In addition to the real RNA-sequencing datasets above, four sets of synthetic RNA-sequencing data were used to compare performances of RNAseqCovarImpute, SI, and CC differential expression analysis. The NSCLC RNA-sequencing data were modified to add known signal using the seqgendiff package [18] (“ Methods”). Compared with fully synthetic count data from theoretical distributions, this method better reflects realistic variability in RNA-sequencing data. Subsets of 25–99% of genes were randomly selected to have their coefficient of association (Log2 fold-changes) with sex set to zero. Distributions of gene expression coefficients associated with sex depended on the desired null gene rates for each synthetic dataset, but followed a similar form compared with the original NSCLC data as intended (Additional file 1: Fig. S5). Coefficients for the remaining genes were drawn randomly from a gamma distribution, and an additional diagnostic confirmed that the coefficients for each gene estimated from the limma-voom pipeline on the synthetic count tables closely matched these pre-defined coefficients input into the seqgendiff package (Additional file 1: Fig. S6). Applied to these synthetic RNA-sequencing datasets, RNAseqCovarImpute had higher TPRs (Additional file 1: Fig. S7) and lower FPRs (Additional file 1: Fig. S8) compared to CC and SI differential expression analysis across most scenarios. Moreover, the advantages of RNAseqCovarImpute were most apparent for synthetic datasets with weaker signals. For example, all methods had FPRs < 0.05 for the synthetic data with the strongest signal (i.e., 75% of genes associated with the predictor of interest and only 25% null genes). However, with 99% null genes and only 1% of genes modified to correlate with the predictor of interest, RNAseqCovarImpute maintained FPRs < 0.05 while the other methods did not (Additional file 1: Fig. S8).

Overall, RNAseqCovarImpute outperformed SI and CC methods in differential expression analysis by achieving higher TPRs, lower FPRs, and lower MAPEs across various real-world and synthetic RNA-sequencing datasets. Its advantages were most notable, especially with respect to controlling FPRs, in scenarios with high levels of missing data or predictors of interest that are only weakly associated with the RNA-sequencing data.

### Computational benchmarks

Methods were benchmarked in an analysis of the ECHO-PATHWAYS dataset with 14,026 genes, 994 observations, 4 covariates in the model, and 55% missingness under MCAR on a Windows machine with 3.8 GHz processing speed and 16 GB random-access memory. MI methods were assessed with 10 imputed datasets. Over three iterations per method, memory allocations and median run times were 16.6 GB and 13.52 min for the RNAseqCovarImpute MI Gene Bin method, 4.32 GB and 2.68 min for the RNAseqCovarImpute MI PCA method, 7.58 GB and 17.46 s for SI, and 3.23 GB and 6.71 s for CC. Computation time was further assessed for the RNAseqCovarImpute MI PCA method over several combinations of sample size, number of genes, and number of imputed datasets (Additional file 1: Fig. S9).

### Application of RNAseqCovarImpute in analysis of maternal age and placental transcriptome

**Fig. 5:**
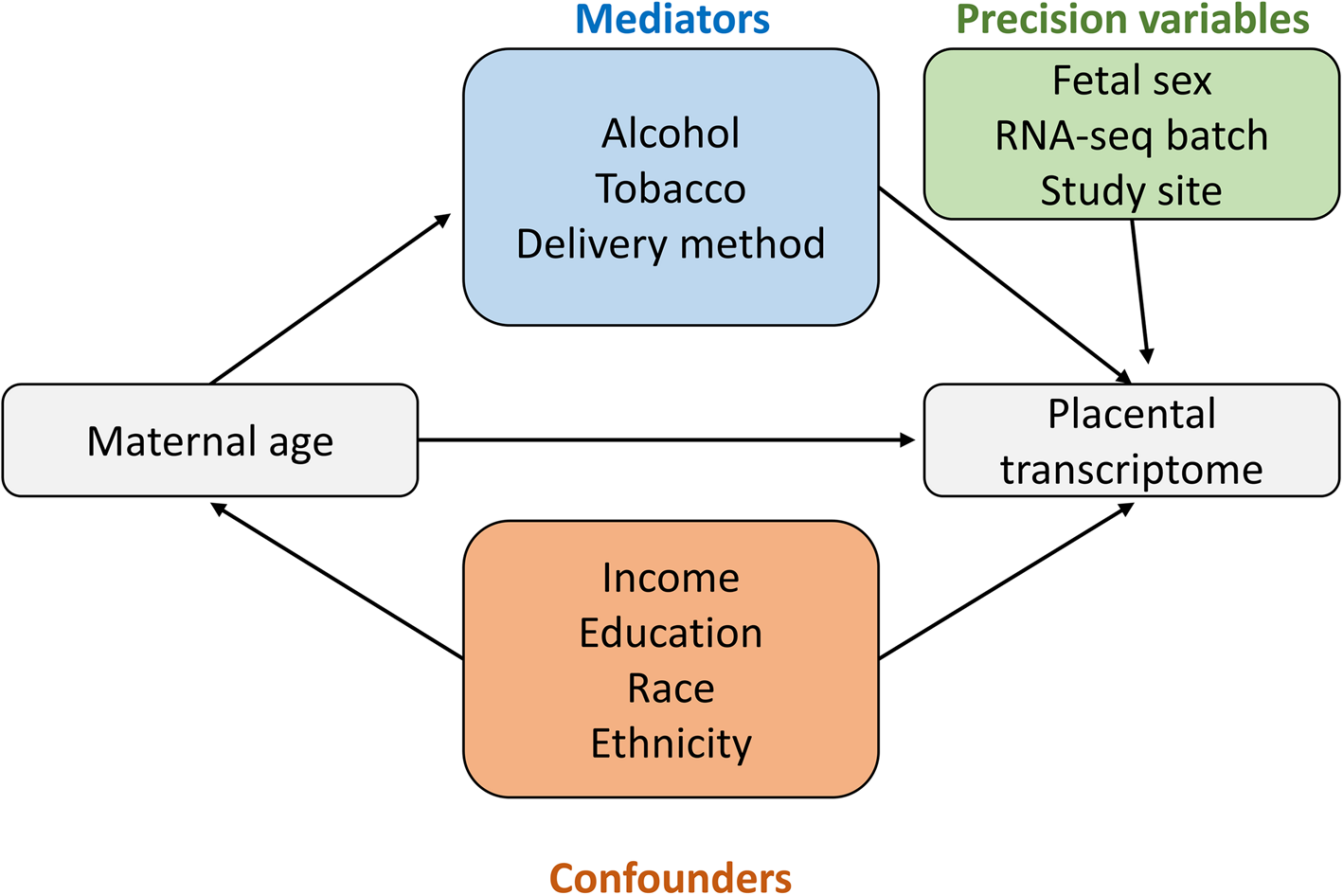
Maternal age and placental transcriptome conceptual model. Conceptual model of association between maternal age (predictor) and the placental transcriptome (outcome). Confounders are upstream causes of both the predictor and outcome. Mediators are on the causal pathway between the predictor and outcome. Precision variables could affect the outcome but have no clear casual effect on the predictor

In a real-world example, RNAseqCovarImpute was applied to the largest placental transcriptomic dataset to-date, which was generated by the ECHO prenatal and early childhood pathways to health (ECHO-PATHWAYS) consortium [5]. This analysis examined the association of maternal age with the placental transcriptome while adjusting for race, ethnicity, family income, maternal education, tobacco and alcohol use during pregnancy, delivery method, study site, fetal sex, and sequencing batch. The causal relationships among these variables are illustrated in Fig. 5.

**Fig. 6:**
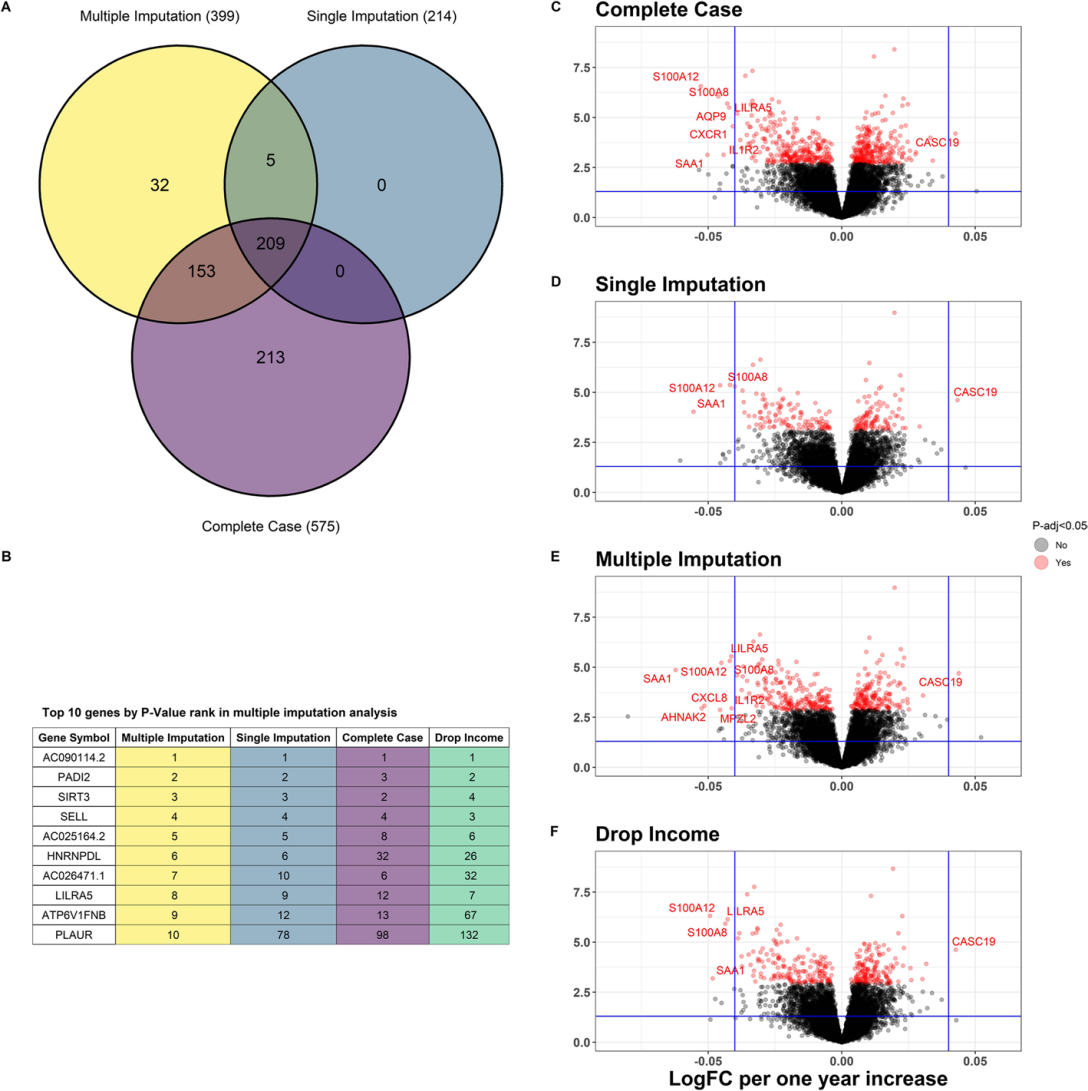
Maternal age and the placental transcriptome differential expression analysis. Venn diagram depicts shared and distinct differentially expressed genes for each method (**A**). *P* value rankings for each method for the top 10 genes with the lowest *P* values from the multiple imputation analysis (**B**). Volcano plots of maternal age associations with placental gene expression in complete case (**C**), single imputation (**D**), and multiple imputation (**E**) analyses. “Drop Income” indicates complete case analysis excluding the income covariate (**F**). Models include the following covariates: maternal race, ethnicity, education, tobacco and alcohol use during pregnancy, household income adjusted for region and inflation, delivery method, fetal sex, sequencing batch, and study site. Log_2_-adjusted fold-changes (LogFCs) shown for each 1 year increase in maternal age. Horizontal and vertical lines at *P* = 0.05 and LogFC ± 0.04, respectively. HGNC gene symbols shown for significant genes with false discovery rate adjusted *P* value (*P*-adj) < 0.05 and LogFC beyond 0.04 cutoff

Among 1045 individuals included in this analysis, 6% (61) were missing data for at least one of the 10 covariates, mostly driven by 4% (41) of individuals missing family income data. There were no missing data for delivery method, study site, fetal sex, and sequencing batch, and a minimally adjusted analysis including these variables identified 1071 differentially expressed genes (DEGs) significantly associated with maternal age. Adjusting for all covariates resulted in fewer maternal age DEGs: in the CC, SI, and RNAseqCovarImpute MI analyses, maternal age was associated with 575, 214, and 399 DEGs, respectively (Fig. 6A). The CC and SI analyses uncovered 91% (362) and 54% (214) of the significant DEGs from the MI method, respectively, while there were 32 DEGs exclusive to MI (Fig. 6A). Additionally, CC analysis was repeated while omitting the family income covariate, which preserved sample size while allowing possible confounding by this variable. This analysis uncovered 334 DEGs, of which 68% (270) overlapped with the DEGs from the MI method (Additional file 1: Fig. S10). Although there were some differences, genes ranked from lowest to highest *P* value followed similar orders between the methods (Fig. 6B). The most substantial differences compared with the *P* value rank order from the MI analysis were observed in the fully adjusted CC and CC omitting family income analyses (Fig. 6B). Imputation diagnostics for family income following the RNAseqCovarImpute MI method indicated good convergence and reasonable imputed values (Additional file 1: Fig. S11).

Many of the top DEGs in the MI analysis, according to their significance and fold-change magnitude (Fig. 6C–F), play roles in inflammatory processes and the immune response. *S100A12* and *S100A8* are pro-inflammatory calcium-, zinc-, and copper-binding proteins, *CXCL8* (IL-8) and *IL1R2* are pro-inflammatory cytokines/cytokine receptors, *SAA1* and *CASC19* are known to be expressed in response to inflammation, while *LILRA5*, a leukocyte receptor gene, may play a role in triggering innate immune responses [19].

**Fig. 7:**
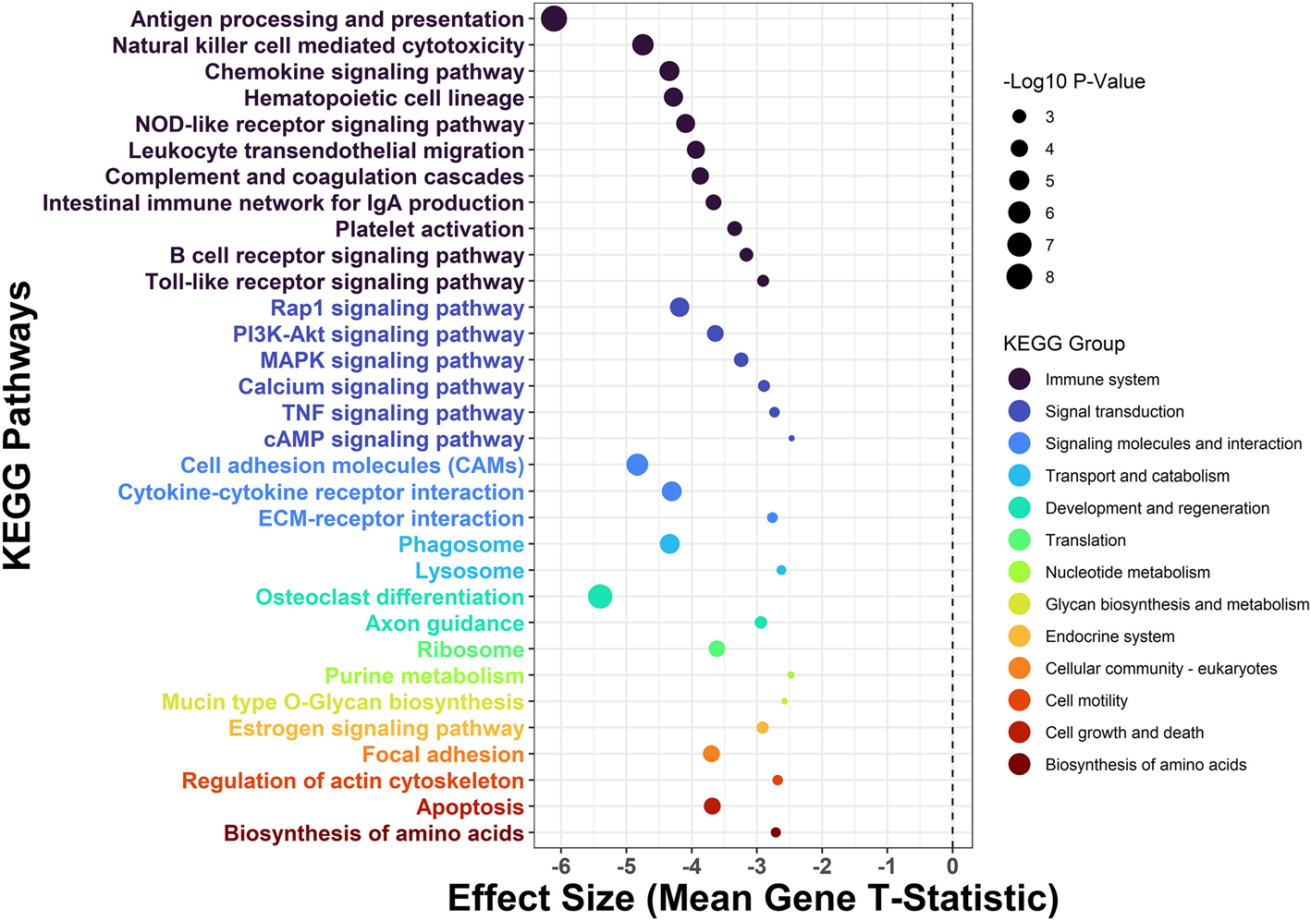
Maternal age and the placental transcriptome pathway analysis. *T*-statistics (Log2FCs divided by standard error) from the differential expression analyses of maternal age were input into pathway analysis for Kyoto Encyclopedia of Genes and Genomes (KEGG) pathways (excluding KEGG human disease pathways) using the generally applicable gene set enrichment (GAGE) method. Mean *t*-statistic of all genes in each KEGG pathway shown with corresponding *P* value from GAGE (larger points indicate smaller *P* values)

Pathway enrichment of the MI differential expression results revealed 32 Kyoto Encyclopedia of Genes and Genomes (KEGG) pathways that were downregulated in association with older maternal age (Fig. 7). Among these downregulated KEGG pathways, 11 belong to the immune system KEGG group and 6 belong to the signal transduction group. Antigen processing and presentation, an immune system KEGG pathway, was the most strongly downregulated pathway according to its enrichment effect size and *P* value (Fig. 7).

### Application of RNAseqCovarImpute in analysis of colorectal carcinoma and the blood platelet transcriptome

In another real-world example, RNAseqCovarImpute was applied to a dataset of blood platelet RNA-sequencing from 42 individuals with colorectal carcinoma and 59 healthy donors [20]. This dataset included 3227 genes after filtering. There were 14 individuals with *KRAS* mutant tumors and 2 individuals with *PIK3CA* mutant tumors compared with 85 wild-type individuals. No data were missing for cancer status or genotype, while 34% (34) were missing data for sex, and 40% (40) were missing data for age. In the CC, SI, and RNAseqCovarImpute MI analyses, colorectal carcinoma was associated with 2491, 2403, and 2579 DEGs, respectively, while controlling for genotype, sex, and age (Additional file 1: Fig. S12A). The CC and SI analyses uncovered 94% (2422) and 92% (2360) of the significant DEGs from the MI method, respectively, while there were 98 DEGs exclusive to MI (Additional file 1: Fig. S12A). Genes ranked from lowest to highest *P* value followed similar orders between the methods (Additional file 1: Fig. S12B).

Many of the top DEGs in the MI analysis, according to their significance and fold-change magnitude (Additional file 1: Fig. S12C–E), have been previously shown to play roles in cancer etiology. For example, colorectal carcinoma was associated with the downregulation of *GK5.* Genes involved in glycerol metabolism, including *GK5*, have been previously implicated in the etiology of cancers, including colorectal carcinoma [21–24]. Mutations in *MDN1*, which was downregulated in association with colorectal cancer here, have been shown to correlate with elevated tumor mutation rates in breast and colorectal cancers [25, 26]. Downregulation of *TNFRSF1B* (a member of the tumor necrosis factor gene family) in association with colorectal cancer here is consistent with prior studies showing lower mRNA expression of *TNFRSF1B* in lung cancer compared with normal lung tissue [27].

## Discussion

We have shown that a MI procedure that includes PCA of the gene expression data in the imputation predictor matrix has a performance advantage relative to CC and SI methods in RNA-sequencing studies using the limma-voom pipeline. We found that our newly implemented MI method in RNAseqCovarImpute was the best performer with higher TPRs and lower FPRs and MAPEs across a wide range of missing data scenarios.

Similar methods allowing for the inclusion of high dimensional outcome data in MI models have been developed for epigenome wide association studies (EWAS) [8, 9]. In EWAS, there appear to be tradeoffs between CC analysis and MI, with MI identifying more true positives but also more false positives [8]. In the simulations presented here, however, RNAseqCovarImpute identified more true DEGs with no false positive tradeoff. The application of MI to EWAS studies represents a more challenging problem owing to higher dimensionality of the methylome (850,000 CpG sites with Illumina’s EPIC array [28]) versus the transcriptome (typically tens of thousands of genes). The application of MI to EWAS studies might benefit from employing a PCA-based approach similar to the one used here. Moreover, future methods development could also tackle the additional challenges in applying MI to alternative epigenomic and transcriptomic methods such as differentially methylated region and pathway enrichment (discussed below) analyses.

To address the sparsity of single-cell RNA-sequencing data, imputation methods to fill in missing or zero RNA-sequencing counts have been extensively developed [29]. Little attention has been paid, however, to the imputation of missing covariate data in studies where gene expression is the outcome of interest. Methods for the treatment of missing data are well-established in observational epidemiology [30], with MI increasingly the method-of-choice [31, 32]. Yet human observational studies of gene expression have often failed to report on the treatment of missing data, despite its prevalence. When missing data are explicitly addressed in this context, researchers typically utilize CC analyses [33–35], while SI is a less common alternative [6]. Despite its advantages, we are unaware of any studies with transcriptomic outcomes that have utilized MI for missing covariate data. The simulations presented here suggest that future observational transcriptomic studies may benefit from employing MI via RNAseqCovarImpute over CC or SI. Moreover, we developed an R package so that users may easily apply the methods presented here to their own data.

We applied RNAseqCovarImpute to a large observational study of maternal age and the placental transcriptome. This analysis assessed the association of maternal age with placental gene expression controlling for confounding variables such as maternal race, ethnicity, and socioeconomic status, and potential mediators such as alcohol and tobacco use during pregnancy. Although there was some overlap, the MI analysis uncovered a different set of differentially expressed genes compared with the CC and SI analyses. CC analysis uncovered a larger number of DEGs compared with MI, possibly indicating that CC may have higher power in some cases. However, the simulations suggest that CC analyses have higher FPRs across many scenarios compared with MI via RNAseqCovarImpute. Individuals with missing data could systematically differ from those with complete data, and dropping these individuals could result in bias. Although higher power of CC remains a possibility, it is more likely that the excess number of DEGs in the CC analysis could be explained by false positives owing to such bias. Ultimately, in a real-world example, the method for dealing with missing data matters, and our simulations suggest that MI should be the preferred approach.

Nevertheless, any of these missing data methods would be a better alternative than omitting covariate control entirely, a common albeit unsatisfactory approach in observational transcriptomic studies. Another reasonable alternative is to perform CC analysis while omitting variables with the most missing data. Compared with fully adjusted CC analysis, CC while omitting the family income variable was more similar to the MI analysis in terms of the total number of DEGs, but displayed more differences in terms of the *P* value rank order of DEGs. Researchers may also opt to only include covariates with complete data, which preserves sample size and avoids the need for imputation but may introduce bias due to uncontrolled variables. For the ECHO-PATHWAYS dataset, only including covariates with complete data resulted in a minimal model adjusting for delivery method, study site, fetal sex, and sequencing batch. This reduced model identified a much larger 1071 DEGs significantly associated with maternal age compared with 214–575 DEGs in the CC, SI, and RNAseqCovarImpute MI analyses adjusting for all covariates. This larger number of DEGs was likely due to confounding by race, ethnicity, lifestyle, and socioeconomic status variables that were not controlled in this analysis. Failure to control for these variables in analyses of maternal age could lead to erroneous conclusions and even faulty clinical recommendations. For instance, studies have shown that the positive associations of young maternal age with child ADHD in unadjusted analyses are eliminated or even reversed following adjustment for confounding and mediating variables [36–38]. Thus, younger pregnancies do not confer increased ADHD risk because of the biology of aging per se but owing to other variables that are correlated with maternal age. Younger mothers are more likely to smoke during pregnancy, and prenatal tobacco exposure may impair neurodevelopment. If the link between younger pregnancy and adverse child development is mediated by increased tobacco exposure, then clinical efforts focusing on reducing tobacco exposures during pregnancy would be more effective than recommendations regarding the ideal age for childbearing [36].

Advanced maternal age is a well-known risk factor for preterm birth [39, 40]. These analyses demonstrated associations of advanced maternal age with downregulation of individual genes (i.e., *CXCL8*) and pathways (i.e., 9 immune system and 4 signal transduction pathways) that were also downregulated in association with spontaneous preterm birth in a prior analysis [41]. Future studies should formally explore these overlapping results as putative mechanistic links between advanced maternal age and preterm birth.

To the best of our knowledge, MI via RNAseqCovarImpute is applicable to any RNA-sequencing study that has missing values in the paired covariate data. In addition to the placental transcriptomics data in ECHO-PATHWAYS, we applied RNAseqCovarImpute to three different datasets. In one example, we analyzed blood platelet RNA-sequencing data from individuals with colorectal carcinoma and healthy controls [20]. This analysis uncovered several differentially expressed genes with known roles in the etiology and progression of various cancers, including colorectal carcinoma. Future studies may utilize RNAseqCovarImpute to achieve higher power and lower FPRs in differential gene expression analyses of a wide range of factors and across diverse tissue types.

One limitation to RNAseqCovarImpute is that it is dependent on selection of a number of PCs to integrate, and the user will need to define the optimal number of PCs to retain using established methods such as Horn’s parallel analysis or a variance explained cutoff. Our testing suggests that several popular methods perform well when missing data is minimal. At high levels of missing data, Horn’s parallel analysis was the best general method of choice, but performance could vary with different datasets. Another drawback to RNAseqCovarImpute is that its compatibility with pathway and gene set enrichment methods is currently limited, as many of these methods were developed without MI in mind. The RNAseqCovarImpute MI method produces one final list of genes with their associated *t*-statistics, log fold changes, and *P* values for differential expression. Thus, the method is compatible with gene set enrichment analyses that utilize gene rankings such as overrepresentation analysis, or gene level statistics such as camera [42] and GAGE (utilized here in the maternal age analysis) [43]. However, the final gene list produced by RNAseqCovarImpute is based on the combined analyses of the MI datasets. Although theoretically possible, methods that require as input a gene expression matrix or data at the individual sample level are likely not out-of-box compatible with RNAseqCovarImpute. Future work could moderate such methods to accommodate analysis of multiply imputed RNA-sequencing data. Additionally, the RNAseqCovarImpute package is also not out-of-box compatible with all differential expression analysis methods, as it was designed to utilize the limma-voom pipeline. Another limitation was that, as an MI method, RNAseqCovarImpute required more processing time compared with CC or SI. Finally, data imputation is a rapidly evolving field, and emerging machine learning SI and MI methods [44] should be tested in the context of RNA-sequencing in future studies.

## Conclusions

As the cost of sequencing decreases, studies of the transcriptome may experience a substantial shift from small-scale in vitro and in vivo experimental systems to larger-scale clinical and epidemiologic contexts where missing covariate data is prevalent. MI is a well-established method to handle missing covariate data in epidemiology, but was previously not compatible with transcriptomic outcome data. We developed an R package, RNAseqCovarImpute, to integrate limma-voom RNA-sequencing analysis with MI for missing covariate data, and demonstrated that this method has superior performance compared with SI and CC analyses. Ultimately, RNAseqCovarImpute represents a promising step towards harmonizing transcriptomic and epidemiologic approaches by addressing the critical need to accommodate missing covariate data in RNA-sequencing studies. Future studies may expand upon the MI methods developed here for RNA-sequencing to address problems associated with missing covariate data in other settings, including DNA methylation, proteomics, metabolomics, and other high dimensional data types.

## Methods

### RNAseqCovarImpute multiple imputation principal component analysis (MI PCA) method

A graphical overview of the RNAseqCovarImpute MI PCA method is shown in Fig. 1, and the corresponding R code are available on GitHub and Bioconductor (see Availability of data and materials).

#### Filtering genes with low counts

A common filtering cutoff in RNA-sequencing is around 10 counts per gene, and filtering on log-counts per million (logCPM) values rather than raw counts is recommended to avoid giving preference to samples with large library sizes [45, 46]. The filtering cutoff on the CPM scale is roughly equal to the count cutoff of 10 divided by the minimum library size in millions. Here, we filter to keep genes with an average CPM across all samples above this cutoff reflecting approximately 10 counts per gene.

#### Data normalization

Following RNA-sequencing and mapping of reads to each gene or probe, an RNA-sequencing dataset consists of a matrix of counts for genes *g* = 1 to the total number of genes *G*, where counts are recorded for all samples *i* = 1 to the total number of samples *n*. The library size *R*_*i*_, which is the total number of reads for a given sample, is expressed as:

$${R}_{i}=\sum_{g=1}^{G}{r}_{gi}$$

The library size *R*_*i*_ is additionally scale-normalized using the weighted trimmed mean of M-values (TMM) method [47], and logCPMs normalized to library size are computed, offsetting the counts by 0.5 to avoid taking the log of zero and offsetting the library size by 1:

$${logCPM}_{gi}={log}_{2}(\frac{{r}_{gi}+0.5}{{R}_{i}+1.0}*{10}^{6})$$

#### Principal component analysis (PCA)

Fitting an imputation model where the number of independent variables is far greater than the number of individuals in the study is generally not feasible. In RNA-sequencing studies with tens of thousands of genes, we can surmount this problem by reducing the dimensionality of the gene expression data with PCA and including a subset of PCs in the MI prediction models.

For the MI PCA method, we conduct PCA using Bioconductor’s PCAtools R package [13] on the gene expression data normalized as described above. There is no universally optimal approach to selecting the number of PCs to retain in PCA. Horn’s parallel analysis retains PCs with eigenvalues greater than eigenvalues of random data [14, 15] and is regarded as one of the best empirical methods to determine component retention in PCA [48]. Performance of MI PCA was compared when using Horn’s parallel analysis, an 80% variance explained cutoff, and the elbow method, where all PCs are retained that come before the elbow point in the curve of variance explained by each successive PC. Methods for determining the number of retained PCs were implemented using Bioconductor’s PCAtools R package [13].

#### Data imputation

The retained PCs are added to the covariate data and utilized along with all covariates in the MI prediction model when creating *m* multiply imputed datasets. Data are imputed using the “mice” R package with its default predictive modeling methods, which are predictive mean matching, logistic regression, polytomous regression, and proportional odds modeling for continuous, binary, categorical, and unordered variables, respectively [49].

#### Differential expression analysis

The limma-voom pipeline is run on each *m* imputed dataset separately. This procedure fits weighted linear models for each gene that take into account individual-level precision weights based on the mean–variance trend [10]. A linear model is fit by ordinary least squares separately for each gene. The model includes an intercept ${\beta }_{0}$, and coefficients ${\beta }_{1}$–${\beta }_{n}$ for any number of covariates ${C}_{1}$–${C}_{n}$.

$${logCPM}_{g}={\beta }_{0}+\sum_{n=1}^{N}{\beta }_{n}*{C}_{n}$$

The geometric mean of the library sizes plus one, $\widetilde R$ is computed. The average logCPM for each gene, $\overline{\log CPM_g}$ is computed and converted to an average log-count by:

$$\widetilde{r}={\overline{logCPM} }_{g}+{log}_{2}\left(\widetilde{R}\right)-{log}_{2}({10}^{6})$$

The regressions provide fitted logCPM values, ${\widehat{\mu }}_{gi}$ for each gene (*g*) and each sample (*i*) that are converted to fitted counts by:

$${{\widehat{\lambda }}_{gi}=\widehat{\mu }}_{gi}+{log}_{2}\left({R}_{i}+1\right)-{log}_{2}({10}^{6})$$

A LOWESS curve is fitted to the square root of the residual standard deviations from the regression models as a function of $\widetilde{r}$, the average log-counts. Interpolating the curve on the interval of library sizes $\widetilde{R}$ defines the piecewise linear function lo() for predicting individual observation-level square-root standard deviations. The predicted square-root standard deviation of individual logCPM observations ${logCPM}_{gi}$ is equal to lo(${\widehat{\lambda }}_{gi}$). Voom precision weights are defined as the predicted inverse variances, lo(${\widehat{\lambda }}_{gi}$)^−4^.

Voom precision weights and logCPM values are input into the limma linear modeling framework which utilizes an empirical Bayes procedure to squeeze gene-wise variances towards a common value [11, 12]. This procedure is run separately on each *m* set of imputed data to obtain coefficients and standard errors for each gene.

#### Pooling results

Rubin’s rules [2] are used to pool coefficients and standard errors, and the Barnard and Rubin adjusted degrees of freedom is calculated [50] (see [3] for more details). From the limma-voom pipeline above, the linear regression coefficient ($\beta$) and the Bayesian moderated standard error (*SE*) for each gene from each *m* number of models on the *m* number of imputed datasets is extracted. The Bayesian moderated degrees of freedom (*df*) are averaged across the *m* models. One gene at a time, results are pooled across the *m* models as follows.

Coefficients are pooled with the basic formula of taking the mean.

$$\overline{\beta }=\frac{1}{m}\sum_{i=1}^{m}{\beta }_{i}$$

Within imputation variance (*V*_*W*_) is the average of the sum of the squared standard errors (*SE*s) divided by *m*.

$${V}_{W}=\frac{1}{m}\sum_{i=1}^{m}{SE}_{i}^{2}$$

Between imputation variance (*V*_*B*_) reflects extra variance due to missing data and is expected to be large when missing data is high. It is calculated as the sum of the squared differences between the pooled coefficient ($\overline{\beta }$) and each coefficient (${\beta }_{i}$) from each imputed dataset divided by *m* − 1.

$${V}_{B}=\frac{{\sum }_{i=1}^{m}{({\beta }_{i}-\overline{\beta })}^{2}}{m-1}$$

Total variance (*V*_*Total*_) is calculated and its square root as the pooled standard error (*SE*_*Pooled*_).

$${V}_{Total}={V}_{W}+{\left(1+\frac{1}{m}\right)V}_{B}$$

$${SE}_{Pooled}=\sqrt{{V}_{Total}}$$

The pooled coefficient ($\overline{\beta }$) divided by the pooled standard error (*SE*_*Pooled*_) is defined as the *t*-statistic (*t*) for significance testing.

$$t=\frac{\overline{\beta }}{{SE}_{Pooled}}$$

The degrees of freedom for significance testing also needs adjustment. First calculate *lambda*, the proportion of total variance due to missingness.

$$lambda= \frac{{V}_{B}+\frac{{V}_{B}}{m}}{{V}_{T}}$$

An older version of the degrees of freedom (*df*_*Old*_) proposed in Rubin (1987) is adjusted using the equations from Barnard and Rubin (1999). This MI adjusted degrees of freedom (*df*_*Adjusted*_) is the same degrees of freedom used in the “mice” R package.

$${df}_{Old}=\frac{m-1}{{lambda}^{2}}$$

$${df}_{Observed}=\frac{df+1}{df+3}*df*(1-lambda)$$

$${df}_{Adjusted}=\frac{{df}_{Old}*{df}_{Observed}}{{df}_{Old}+{df}_{Observed}}$$

*P* values are derived from the *t*-distribution. In R, 2-sided *P* values can be calculated using the *pt* function, which returns the area for the Student’s *t*-distribution to the left of the *t*-statistic for a given degrees of freedom. In R:

$$2*pt(-abs\left(t\right), {df}_{Adjusted})$$

After this pooling procedure is completed for every gene, *P* values for the linear model contrast of interest are adjusted for false-discovery-rate control [51].

### Performance on three example datasets

Performance was evaluated in a simulation study using three real RNA-sequencing and covariate datasets and four synthetic sets of RNA-sequencing data (described below). Performance was compared between SI followed by the standard limma-voom differential expression analysis, CC limma-voom differential expression analysis, and the two RNAseqCovarImpute methods, MI Gene Bin (Additional file 1: Supplemental Methods) and MI PCA (described above).

#### Determining true differentially expressed genes (DEGs)

Differential expression analysis using the limma-voom pipeline was conducted on the entire set of observations with their complete covariate data (hereinafter “full data”). These models estimated the effect of a predictor of interest on gene expression while controlling for several covariates. Genes significantly associated with the predictor of interest at FDR < 0.05 in these full data models were defined as true DEGs.

#### Simulating missing data under different missingness mechanisms

Missingness was simulated using the ampute function from the “mice” package [49]. Missingness was simulated to emulate a common situation in scientific research where an investigator has complete data for a predictor of interest, but may have missing data for other important covariates. Therefore, missingness was only induced in adjustment covariates and not the predictor of interest. We explored scenarios with various levels of missing data ranging from 5 to 85% of participants having at least one missing data point, and under three missingness mechanisms: missing completely at random (MCAR), missing at random (MAR), and missing not at random (MNAR). We simulated ten datasets for each missingness mechanism at each level of missingness before applying the SI, CC, MI, and MI PCA methods and comparing the results with the full data model.

One or two covariates (described in detail below for each dataset) were defined as MNAR variables: these variables were not included as adjustment covariates in the differential expression analysis, but had influence in determining the missingness in the other covariate data. Under the MAR mechanism, the data that explain the missingness are all available. Thus, for the MAR mechanism, the SI, MI, and MI PCA methods had access to these MNAR variables while imputing missing covariate data. Under the MNAR mechanism, patterns of missingness in the data are related to unobserved or unmeasured factors. Thus, for the MNAR mechanism, the SI, MI, and MI PCA methods did not have access to these MNAR variables while imputing missing covariate data. Under the MCAR mechanism, missingness in the data are completely random and do not depend on values of the covariates.

CC analyses dropped any individual with at least one missing data point, while SI imputed missing data using the missForest package [52]. The limma-voom pipeline was applied for CC and SI as described for the full data model.

#### Evaluating results

Our objective was to evaluate the ability of the SI, CC, MI, and MI PCA methods to identify true DEGs from the full data model as significant while limiting false positives. True DEGs from the full data model that were also identified as significant by a given method were defined as true positives. We report the true positive rate (TPR) as the proportion of true DEGs identified as significant for each method out of the total number of true DEGs from the full data model. Genes erroneously identified as significant by a given method that were not true DEGs from the full data analysis were defined as false positives. We report the false positive rate (FPR) as the proportion of false positives out of the total number of significant results for each method. We report the mean absolute percentage error (*MAPE*) across all true DEGs to characterize the ability of each method to reproduce gene expression coefficients from the full data model, where ${\beta }_{truth g}$ is defined as the true coefficient from a DEG in the full data model, and ${\beta }_{g}$ is defined as the coefficient for the same gene estimated using SI, CC, MI, or MI PCA following simulated missingness. *MAPE* was calculated as:

$$MAPE=\frac{1}{G}\sum_{g=1}^{G}\frac{\left|{\beta }_{truth g}-{\beta }_{g}\right|}{\left|{\beta }_{truth g}\right|}$$

#### Datasets

Three RNA-sequencing datasets with mapped reads were obtained and processed as described above in “ Data normalization” and “ Filtering genes with low counts.” The first dataset was based on placental RNA-sequencing and covariate data from the ECHO prenatal and early childhood pathways to health (ECHO-PATHWAYS) consortium [5]. This study harmonized extant data from three pregnancy cohorts from diverse populations across the country. The consortium’s core aim is to explore the impact of chemical exposures and psychosocial stressors experienced by the mother during pregnancy on child development, and to assess potential underlying placental mechanisms. To investigate placental mechanisms, the study generated RNA-sequencing data for the CANDLE and GAPPS pregnancy cohort samples. All participants of the CANDLE and GAPPS studies provided informed consent upon enrollment and research protocols were approved by the Institutional Review Boards (IRBs) at the University of Tennessee Health Science Center (IRB approval: 17–05154-XP) as well as the Seattle Children’s Hospital (IRB approval: STUDY00000608) and the University of Washington (IRB approval: STUDY00000638). The generation of placental RNA-sequencing data for this study is described elsewhere [41]. Among the enrolled study sample of 1503, transcriptomic data are available for 1083 individuals. We excluded 18 placental abruptions and 20 individuals missing maternal age data, leaving a sample of 1045. We retained only protein-coding genes, processed pseudogenes, and lncRNAs.

Covariates from the ECHO-PATHWAYS dataset included in the simulation study were maternal age (continuous), child sex (male versus female), RNA-sequencing batch, maternal tobacco use during pregnancy (yes versus no), maternal alcohol use during pregnancy (yes versus no), and family income (continuous). The full data model restricted to 994 individuals with complete data for these variables. Mothers self-reported alcohol use, while the positive tobacco exposure group included individuals with maternal urine cotinine above 200 ng/mL [53], as well as individuals who were below this cutoff but self-reported tobacco use during pregnancy. Maternal age was defined as the predictor of interest, while sex, prenatal tobacco exposure, and RNA-sequencing batch were modeled as covariates. Simulated missing data ranged from 5 to 55% of participants having at least one missing data point. Maternal alcohol use and family income served as MNAR variables. Levels of missingness according to different values of these MNAR variables were summarized to illustrate differences between MAR, MNAR, and MCAR missingness mechanisms.

Two additional datasets were selected based on their large sample sizes, public availability, and ample number of covariates that could be examined in covariate imputation analyses. The non-small cell lung cancer (NSCLC) dataset was downloaded from the European Molecular Biology Laboratory—European Bioinformatics Institute (EMBL-EBI: E-GEOD-81089) and is based on [16]. For the NSCLC dataset (*N* = 670), the association of sex (male versus female) with the transcriptome was examined, adjusting for participant age (continuous) and participant smoking status (smoker versus ex-smoker versus non-smoker). Sampling site (tumor versus non-malignant) served as an MNAR variable. The Epstein-Barr virus (EBV) dataset was downloaded from EMBL-EBI (E-MTAB-7805) and is based on [17]. For the EBV dataset (*N* = 384), the association of time (continuous days) with the transcriptome was examined, adjusting for infection status (EBV infected versus not infected). Donor source (categorical, three individuals) served as an MNAR variable. All methods performed better at recovering the full data model results for the EMBL-EBI datasets compared with the ECHO-PATHWAYS dataset, so analyses with these datasets examined 55–85% of participants having at least one missing data point.

Finally, four sets of synthetic RNA-sequencing data were also used to compare performances of RNAseqCovarImpute (MI PCA Horn method), SI, and CC differential expression analysis. The NSCLC RNA-sequencing data were modified to add known signal using the seqgendiff package [18]. The method relies on binomial thinning of the RNA-sequencing count matrix to closely match user defined coefficients. Rather than generating counts from theoretical distributions, thinning a real set of RNA-sequencing counts can better preserve realistic variability and inter-gene correlations typical of RNA-sequencing data [18]. Subsets of 25%, 50%, 75%, or 99% of genes were randomly selected to have their coefficient of association (Log2 fold-changes) with sex set to zero. The remaining coefficients were drawn randomly from a gamma distribution generated using rgamma(shape = 1) from the stats package in R.

### Application of RNAseqCovarImpute in analysis of maternal age and the placental transcriptome

This analysis examined the association of maternal age with the placental transcriptome while controlling for 10 covariates using the ECHO-PATHWAYS sample described above (*N* = 1045). Covariates included family income adjusted for region and inflation (USD), maternal race (Black vs. other), maternal ethnicity (Hispanic/Latino vs. not Hispanic/Latino), maternal education (< high school vs. high school completion vs. college or technical school vs. graduate/professional degree), study site, maternal alcohol during pregnancy (yes vs. no), maternal tobacco during pregnancy (yes vs. no), delivery method (vaginal vs. C-section), fetal sex (male vs. female), and RNA-sequencing batch. The causal relationships among these variables are illustrated in Fig. 5. For the maternal race variable, American Indian/Alaska Native, multiple race, and other were collapsed along with White participants to avoid small or zero cell sizes in multivariable models. Only protein-coding genes, processed pseudogenes, and lncRNAs, and genes with average log-CPM > 0 (approximately 10 counts for this dataset) were retained, resulting in a final sample of 14,029 genes. DEGs associated with maternal age while adjusting for all 10 covariates were compared between the CC, SI, and RNAseqCovarImpute MI PCA methods. To retain the entire sample size without covariate imputation, a reduced model was fit by omitting any covariates with missing data. Additionally, an alternative CC analysis was performed while omitting family income, the variable with the most missing data.

*T*-statistics (Log2FCs divided by standard error) from the differential expression analyses were input into pathway analysis for Kyoto Encyclopedia of Genes and Genomes (KEGG) pathways (excluding KEGG human disease pathways) using the generally applicable gene set enrichment (GAGE) method [43]. For pathways with GAGE FDR < 0.05, GAGE *P* values and the mean differential expression *t*-statistic for all genes in the pathway were plotted.

### Application of RNAseqCovarImpute in analysis of colorectal carcinoma and the blood platelet transcriptome

In another real-world example, RNAseqCovarImpute was applied to a dataset of blood platelet RNA-sequencing from 42 individuals with colorectal carcinoma and 59 healthy donors (EMBL-EBI: E-GEOD-68086) [20]. This analysis examined the association of colorectal carcinoma versus healthy cancer status with the transcriptome while controlling for genotype (*KRAS* vs. *PIK3CA* vs. wild-type), sex, and age (*N* = 101). DEGs associated with colorectal carcinoma while adjusting for these covariates were compared between the CC, SI, and RNAseqCovarImpute MI PCA methods.

### Supplementary Information

Additional file 1: Supplemental Methods, Supplemental Results, and Supplemental Figs. S1–S12.

Additional file 2. The review history.

## Data Availability

The RNAseqCovarImpute R package is available at the Bioconductor repository under the GNU General Public License v3.0 (10.18129/B9.bioc.RNAseqCovarImpute) [54]. Source code for simulating missing covariate data and evaluating different methods for handling missing data, including the complete case, single imputation, and RNAseqCovarImpute methods, is available at the Zenodo repository under the GNU General Public License v3.0 (10.5281/zenodo.13314514) [55]. The NSCLC data are available at EMBL-EBI accession E-GEOD-81089 [16]. The EBV data are available at EMBL-EBI accession E-MTAB-7805 [17]. The colorectal carcinoma data are available at EMBL-EBI accession E-GEOD-68086 [20]. The ECHO-PATHWAYS RNA-sequencing data are available at dbGaP study accessions phs003619.v1.p1 and phs003620.v1.p1. The ECHO-PATHWAYS covariate data are available upon reasonable request following the data sharing guidelines of the ECHO-PATHWAYS consortium, outlined in LeWinn et al. [5].

## References

[1] van Buuren S. Flexible Imputation of Missing Data. Second Edition (2nd ed.). Chapman and Hall/CRC; 2018. 10.1201/9780429492259.

[2] Rubin DB. Multiple Imputation for Nonresponse in Surveys. New York: Wiley; 2004.

[3] Heymans M, Eekhout I. Applied missing data analysis with SPSS and (R) Studio. Heymans and Eekhout: Amsterdam, The Netherlands: 20 Available online: https://bookdown/org/mwheymans/bookmi/. 2019. Accessed 23 May 2020.

[4] Goodwin S, McPherson JD, McCombie WR. Coming of age: ten years of next-generation sequencing technologies. Nat Rev Genet. 2016;17(6):333–51. 27184599 10.1038/nrg.2016.49 PMC10373632

[5] LeWinn KZ, Karr CJ, Hazlehurst M, Carroll K, Loftus C, Nguyen R, et al. Cohort profile: the ECHO prenatal and early childhood pathways to health consortium (ECHO-PATHWAYS). BMJ Open. 2022;12(10):e064288. 36270755 10.1136/bmjopen-2022-064288 PMC9594508

[6] Eaves LA, Bulka CM, Rager JE, Gardner AJ, Galusha AL, Parsons PJ, et al. Metal mixtures modeling identifies birth weight-associated gene networks in the placentas of children born extremely preterm. Chemosphere. 2023;313:137469. 36493891 10.1016/j.chemosphere.2022.137469 PMC10476282

[7] Little RJ. Regression with missing X’s: a review. J Am Stat Assoc. 1992;87(420):1227–37.

[8] Mills HL, Heron J, Relton C, Suderman M, Tilling K. Methods for dealing with missing covariate data in epigenome-wide association studies. Am J Epidemiol. 2019;188(11):2021–30. 31504104 10.1093/aje/kwz186 PMC6825836

[9] Wu C, Demerath EW, Pankow JS, Bressler J, Fornage M, Grove ML, et al. Imputation of missing covariate values in epigenome-wide analysis of DNA methylation data. Epigenetics. 2016;11(2):132–9. 26890800 10.1080/15592294.2016.1145328 PMC4846117

[10] Law CW, Chen Y, Shi W, Smyth GK. voom: precision weights unlock linear model analysis tools for RNA-seq read counts. Genome Biol. 2014;15(2):1–17. 10.1186/gb-2014-15-2-r29 PMC4053721 24485249

[11] Ritchie ME, Phipson B, Wu D, Hu Y, Law CW, Shi W, et al. limma powers differential expression analyses for RNA-sequencing and microarray studies. Nucleic Acids Res. 2015;43(7):e47-e. 25605792 10.1093/nar/gkv007 PMC4402510

[12] Smyth GK. Linear models and empirical Bayes methods for assessing differential expression in microarray experiments. Statistical applications in genetics and molecular biology. 2004;3(1). 10.2202/1544-6115.1027. 10.2202/1544-6115.1027 16646809

[13] Blighe K, Lun A. PCAtools: PCAtools: Everything Principal Components Analysis. R package version 2.16.0; 2024. https://github.com/kevinblighe/PCAtools.

[14] Buja A, Eyuboglu N. Remarks on parallel analysis. Multivar Behav Res. 1992;27(4):509–40. 10.1207/s15327906mbr2704_2 26811132

[15] Horn JL. A rationale and test for the number of factors in factor analysis. Psychometrika. 1965;30:179–85. 14306381 10.1007/BF02289447

[16] Djureinovic D, Hallström BM, Horie M, Mattsson JSM, La Fleur L, Fagerberg L, et al. Profiling cancer testis antigens in non–small-cell lung cancer. JCI Insight. 2016;1(10). https://doi.org/10.1172%2Fjci.insight.86837. 10.1172/jci.insight.86837 PMC5033889 27699219

[17] Mrozek-Gorska P, Buschle A, Pich D, Schwarzmayr T, Fechtner R, Scialdone A, et al. Epstein-Barr virus reprograms human B lymphocytes immediately in the prelatent phase of infection. Proc Natl Acad Sci. 2019;116(32):16046–55. 31341086 10.1073/pnas.1901314116 PMC6690029

[18] Gerard D. Data-based RNA-seq simulations by binomial thinning. BMC Bioinformatics. 2020;21:1–14. 32448189 10.1186/s12859-020-3450-9 PMC7245910

[19] UniProt: the universal protein knowledgebase in 2023. Nucleic Acids Research. 2023;51(D1):D523–D31. 10.1093/nar/gkac1052. 10.1093/nar/gkac1052 PMC9825514 36408920

[20] Best MG, Sol N, Kooi I, Tannous J, Westerman BA, Rustenburg F, et al. RNA-Seq of tumor-educated platelets enables blood-based pan-cancer, multiclass, and molecular pathway cancer diagnostics. Cancer Cell. 2015;28(5):666–76. 26525104 10.1016/j.ccell.2015.09.018 PMC4644263

[21] Liu Y, Zhou F, Yang H, Zhang Z, Zhang J, He K, et al. Porphyromonas gingivalis promotes malignancy and chemo-resistance via GSK3β-mediated mitochondrial oxidative phosphorylation in human esophageal squamous cell carcinoma. Transl Oncol. 2023;32:101656. 36989676 10.1016/j.tranon.2023.101656 PMC10074990

[22] Liu X-S, Chen Y-X, Wan H-B, Wang Y-L, Wang Y-Y, Gao Y, et al. TRIP6 a potential diagnostic marker for colorectal cancer with glycolysis and immune infiltration association. Sci Rep. 2024;14(1):4042. 38369589 10.1038/s41598-024-54670-0 PMC10874967

[23] Nishida N, Nagahara M, Sato T, Mimori K, Sudo T, Tanaka F, et al. Microarray analysis of colorectal cancer stromal tissue reveals upregulation of two oncogenic miRNA clusters. Clin Cancer Res. 2012;18(11):3054–70. 22452939 10.1158/1078-0432.CCR-11-1078

[24] Lee CJ, Baek B, Cho SH, Jang TY, Jeon SE, Lee S, et al. Machine learning with in silico analysis markedly improves survival prediction modeling in colon cancer patients. Cancer Med. 2023;12(6):7603–15. 36345155 10.1002/cam4.5420 PMC10067044

[25] Liu Z, Zhang Y, Dang Q, Wu K, Jiao D, Li Z, et al. Genomic alteration characterization in colorectal cancer identifies a prognostic and metastasis biomarker: FAM83A| IDO1. Front Oncol. 2021;11:632430. 33959500 10.3389/fonc.2021.632430 PMC8093579

[26] Hao S, Huang M, Xu X, Wang X, Huo L, Wang L, et al. MDN1 mutation is associated with high tumor mutation burden and unfavorable prognosis in breast cancer. Front Genet. 2022;13:857836. 35386280 10.3389/fgene.2022.857836 PMC8978890

[27] Guo Y, Feng Y, Liu H, Luo S, Clarke JW, Moorman PG, et al. Potentially functional genetic variants in the TNF/TNFR signaling pathway genes predict survival of patients with non-small cell lung cancer in the PLCO cancer screening trial. Mol Carcinog. 2019;58(7):1094–104. 30989732 10.1002/mc.23017 PMC6548610

[28] Moran S, Arribas C, Esteller M. Validation of a DNA methylation microarray for 850,000 CpG sites of the human genome enriched in enhancer sequences. Epigenomics. 2016;8(3):389–99. 26673039 10.2217/epi.15.114 PMC4864062

[29] Hou W, Ji Z, Ji H, Hicks SC. A systematic evaluation of single-cell RNA-sequencing imputation methods. Genome Biol. 2020;21:1–30. 10.1186/s13059-020-02132-x PMC7450705 32854757

[30] Lee KJ, Tilling KM, Cornish RP, Little RJ, Bell ML, Goetghebeur E, et al. Framework for the treatment and reporting of missing data in observational studies: the treatment and reporting of missing data in observational studies framework. J Clin Epidemiol. 2021;134:79–88. 33539930 10.1016/j.jclinepi.2021.01.008 PMC8168830

[31] Mackinnon A. The use and reporting of multiple imputation in medical research–a review. J Intern Med. 2010;268(6):586–93. 20831627 10.1111/j.1365-2796.2010.02274.x

[32] Hayati Rezvan P, Lee KJ, Simpson JA. The rise of multiple imputation: a review of the reporting and implementation of the method in medical research. BMC Med Res Methodol. 2015;15:1–14. 25880850 10.1186/s12874-015-0022-1 PMC4396150

[33] Enquobahrie DA, MacDonald J, Hussey M, Bammler TK, Loftus CT, Paquette AG, et al. Prenatal exposure to particulate matter and placental gene expression. Environ Int. 2022;165:107310. 35653832 10.1016/j.envint.2022.107310 PMC9235522

[34] Paquette AG, MacDonald J, Lapehn S, Bammler T, Kruger L, Day DB, et al. A comprehensive assessment of associations between prenatal phthalate exposure and the placental transcriptomic landscape. Environ Health Perspect. 2021;129(9):097003. 34478338 10.1289/EHP8973 PMC8415559

[35] Paquette AG, Lapehn S, Freije S, MacDonald J, Bammler T, et al. Placental transcriptomic signatures of prenatal exposure to Hydroxy-Polycyclic aromatic hydrocarbons. Environ Int. 2023;172:107763. 10.1016/j.envint.2023.107763 PMC10211546 36689866

[36] Baker BH, Joo YY, Park J, Cha J, Baccarelli AA, Posner J. Maternal age at birth and child attention-deficit hyperactivity disorder: causal association or familial confounding? J Child Psychol Psychiatry. 2023;64(2):299–310. 36440655 10.1111/jcpp.13726

[37] Hvolgaard Mikkelsen S, Olsen J, Bech BH, Obel C. Parental age and attention-deficit/hyperactivity disorder (ADHD). Int J Epidemiol. 2017;46(2):409–20. 27170763 10.1093/ije/dyw073

[38] Chang Z, Lichtenstein P, D’Onofrio BM, Almqvist C, Kuja-Halkola R, Sjölander A, et al. Maternal age at childbirth and risk for ADHD in offspring: a population-based cohort study. Int J Epidemiol. 2014;43(6):1815–24. 25355726 10.1093/ije/dyu204 PMC4276066

[39] Waldenström U, Cnattingius S, Vixner L, Norman M. Advanced maternal age increases the risk of very preterm birth, irrespective of parity: a population-based register study. BJOG: An International Journal of Obstetrics and Gynaecology. 2017;124(8):1235–44. 27770495 10.1111/1471-0528.14368

[40] Fuchs F, Monet B, Ducruet T, Chaillet N, Audibert F. Effect of maternal age on the risk of preterm birth: a large cohort study. PLoS One. 2018;13(1):e0191002. 29385154 10.1371/journal.pone.0191002 PMC5791955

[41] Paquette AG, MacDonald J, Bammler T, Day DB, Loftus CT, Buth E, et al. Placental transcriptomic signatures of spontaneous preterm birth. Am J Obs Gynecol. 2023;228(1):73 e1-. e18. 10.1016/j.ajog.2022.07.015 PMC9790028 35868418

[42] Wu D, Smyth GK. Camera: a competitive gene set test accounting for inter-gene correlation. Nucleic acids research. 2012;40(17):e133-e. 22638577 10.1093/nar/gks461 PMC3458527

[43] Luo W, Friedman MS, Shedden K, Hankenson KD, Woolf PJ. GAGE: generally applicable gene set enrichment for pathway analysis. BMC Bioinformatics. 2009;10:1–17. 19473525 10.1186/1471-2105-10-161 PMC2696452

[44] Luo Y. Evaluating the state of the art in missing data imputation for clinical data. Briefings in Bioinformatics. 2022;23(1):bbab489. 34882223 10.1093/bib/bbab489 PMC8769894

[45] Law CW, Alhamdoosh M, Su S et al. RNA-seq analysis is easy as 1-2-3 with limma, Glimma and edgeR [version 3; peer review: 3 approved]. F1000Res. 2018;5:1408. 10.12688/f1000research.9005.3. 10.12688/f1000research.9005.1 PMC4937821 27441086

[46] Chen Y, Lun ATL and Smyth GK. From reads to genes to pathways: differential expression analysis of RNA-Seq experiments using Rsubread and the edgeR quasi-likelihood pipeline [version 2; peer review: 5 approved]. F1000Res. 2016;5:1438. 10.12688/f1000research.8987.2. 10.12688/f1000research.8987.1 PMC4934518 27508061

[47] Robinson MD, Oshlack A. A scaling normalization method for differential expression analysis of RNA-seq data. Genome Biol. 2010;11(3):1–9. 10.1186/gb-2010-11-3-r25 PMC2864565 20196867

[48] Dinno A. Exploring the sensitivity of Horn’s parallel analysis to the distributional form of random data. Multivar Behav Res. 2009;44(3):362–88. 10.1080/00273170902938969 PMC2838619 20234802

[49] Van Buuren S, Groothuis-Oudshoorn K. mice: multivariate imputation by chained equations in R. J Stat Softw. 2011;45:1–67. 10.18637/jss.v045.i03

[50] Barnard J, Rubin DB. Miscellanea. Small-sample degrees of freedom with multiple imputation. Biometrika. 1999;86(4):948–55. 10.1093/biomet/86.4.948

[51] Benjamini Y, Hochberg Y. Controlling the false discovery rate: a practical and powerful approach to multiple testing. J Roy Stat Soc B. 1995;57(1):289–300. 10.1111/j.2517-6161.1995.tb02031.x

[52] Stekhoven DJ, Bühlmann P. MissForest—non-parametric missing value imputation for mixed-type data. Bioinformatics. 2012;28(1):112–8. 22039212 10.1093/bioinformatics/btr597

[53] Schick SF, Blount BC, Jacob P 3rd, Saliba NA, Bernert JT, El Hellani A, et al. Biomarkers of exposure to new and emerging tobacco delivery products. Am J Physiol Lung Cell Mol Physiol. 2017;313(3):L425–52. 28522563 10.1152/ajplung.00343.2016 PMC5626373

[54] Baker BH, Sathyanarayana S, Szpiro AA, MacDonald JW, Paquette AG. RNAseqCovarImpute: impute covariate data in RNA sequencing studies. R package version 1.2.0. 2024. 10.18129/B9.bioc.RNAseqCovarImpute.

[55] Baker BH. 2024. RNAseqCovarImpute source code for NSCLC data analysis. 10.5281/zenodo.13314514.

