# Supplementary material for: RNAseqCovarImpute: a multiple imputation procedure that outperforms complete case and single imputation differential expression analysis
Source: Genome Biol. 2024 Sep 3;25:236. doi: 10.1186/s13059-024-03376-7 (PMC11370143; doi:10.1186/s13059-024-03376-7)
Supplement: Supplementary file 1 — Additional file 1: Supplemental Methods, Supplemental Results, and Supplemental Figs. S1–S12. [file 13059_2024_3376_MOESM1_ESM.docx]

**Supplemental Methods**

**Multiple Imputation by Gene Bin Method**

Binning genes

Fitting an imputation model where the number of independent variables is far greater than the number of individuals in the study is generally not feasible. In RNA-sequencing studies with tens of thousands of genes, we can surmount this problem by reducing the dimensionality of the gene expression data with PCA. Another option is to bin genes into smaller groups, create MI datasets for each gene bin, and conduct differential expression analyses on each bin separately.

In our MI Gene Bin method (Fig. S1), the default bin size is approximately 1 gene per 10 individuals in the study, but the user can specify a different ratio. For example, in a study with 500 participants and 10,000 genes, 200 bins of 50 genes would be created using the default ratio. If the total number of genes is not divisible by the bin size, the method flexibly creates bins of different sizes. The order of the features (e.g., ENSEMBL gene identifiers) should be randomized before binning.

Data imputation

Data are imputed using the mice R package with its default predictive modeling methods, which are predictive mean matching, logistic regression, polytomous regression, and proportional odds modeling for continuous, binary, categorical, and unordered variables, respectively (Van Buuren & Groothuis-Oudshoorn, 2011). The user may specify “*m*”, the number of imputed datasets, and “*maxit*”, the number of iterations for each imputation. *m* imputed datasets are created separately for each gene bin, where the imputation predictor matrix includes all covariates along with the log-CPM for all the genes in a particular bin. Thus, each gene bin contains *m* sets of imputed data. In our example scenario above with 200 bins of 50 genes, 200 separate MI datasets will be created (one MI dataset for each bin), and the MI prediction models for each bin will include all covariates along with the log-CPM gene expression data for 50 genes.

Differential expression analysis

DEGs are determined via the limma-voom pipeline. This procedure fits weighted linear models for each gene that take into account individual-level precision weights based on the mean-variance trend (Law, Chen, Shi, & Smyth, 2014). Model results are further moderated with the limma empirical Bayes procedure in which gene-wise variances are squeezed towards a global mean-variance trend curve (Ritchie et al., 2015; Smyth, 2004).

Limma-voom cannot run naively across the separate datasets for each bin of genes, as a core principle of limma-voom is borrowing strength across the entire gene expression set. To surmount this issue, our method first constructs the mean-variance curve using all genes within all m imputations. Gene-wise linear models are fit using the lmFit function taking into account the experimental design, with all covariates, separately within each gene bin on each *m* imputed dataset. The residual standard deviations and average log-counts are extracted from all lmFit models across all *m* imputations to fit the robust LOWESS curve that is used to estimate voom precision weights (Law et al., 2014).

We apply a modified voom function followed by the lmFit function from the limma package (Ritchie et al., 2015; Smyth, 2004) separately within each gene bin on each *m* imputed dataset to estimate precision weights and fit weighted linear models for every gene. We modified the voom function to allow input of bins of outcome genes while utilizing the above precision weights, which were calculated using all genes across all *m* imputations, rather than estimating precision weights separately within each gene bin. Genes are then un-binned, and the *m* sets of lmFit model results are stacked into *m* tables of model results. After un-binning, each *m* table contains a set of model results for all genes in the analysis. The squeezeVar function from the limma package is then used separately on each *m* set of lmFit model results to apply the limma empirical Bayes procedure and estimate prior variances and degrees of freedom.

**Supplemental Results**

We compared the Gene Bin method for multiple imputation (MI) differential expression analysis (MI Gene Bin) with the principal component analysis (PCA) method, both implemented in the RNAseqCovarImpute package. For the PCA approach, we additionally tested three methods for determining the number of PCs to retain: Horn’s parallel analysis (MI PCA Horn) an 80% variation explained cutoff (MI PCA 80%), and the elbow method (MI PCA Elbow), where all PCs are retained that come before the elbow point in the curve of variance explained by each successive PC.

For the ECHO-PATHWAYS dataset, MI PCA Horn retained 35 PCs, MI PCA 80% retained 213 PCs, and MI PCA Elbow retained 15 PCs. The MI PCA Horn, MI PCA Elbow, and MI Gene Bin methods had similar true positive rates (TPRs), while the MI PCA 80% method had slightly higher TPRs (Fig. S2A). The MI PCA Horn method had the lowest false positive rates (FPRs), while the MI Gene Bin method FPRs were generally controlled at approximately 0.05 (Fig. S2B). The MI PCA 80% and MI PCA Elbow methods, on the other hand, resulted in FPRs>0.05 in many scenarios (Fig. S2B). The MI PCA Horn method also had the lowest mean absolute percentage error (MAPE) across most scenarios (Fig. S2C).

For the NSCLC dataset, MI PCA Horn retained 56 PCs, MI PCA 80% retained 47 PCs, and MI PCA Elbow retained 12 PCs. All PCA methods had similarly high TPRs, while the MI Gene Bin method had generally lower TPRs (Fig. S3A). All four methods performed similarly with respect to FPR and MAPE (Fig. S3 B and C).

For the EBV dataset, MI PCA Horn retained 10 PCs, while MI PCA 80% retained 7 PCs. The elbow method also retained 10 PCs and thus was not tested (as the results were identical with the Horn method). All RNAseqCovarImpute methods performed extremely well on this dataset, with TPRs of 1 as well as FPRs and MAPES of 0 across many scenarios (Fig. S4). However, when 85% of participants had at least one missing data point, performance of the MI Gene Bin method was poor with respect to TPR across many simulations (Fig. S4).

The NSCLC and EBV datasets were less variable, requiring fewer PCs to explain maximal variance compared with the ECHO-PATHWAYS dataset. Consequentially, a similar number of PCs was retained using Horn’s parallel analysis and the 80% variance explained cutoff for those datasets. For the ECHO-PATHWAYS dataset, however, many more PCs (213) were retained using the 80% variance cutoff compared with parallel analysis (35). For the ECHO-PATHWAYS dataset, the MI PCA 80% method had slightly higher TPRs, while MI PCA Horn had better false discovery control. Furthermore, including a large number of variables in a statistical model is generally not recommended. Avoiding this problem is a core goal of RNAseqCovarImpute, and defaulting to Horn’s parallel analysis over an explained variance cutoff can facilitate this goal. In a dataset of approximately 1,000 participants, adding 213 PCs as variables to the MI prediction model is not trivial. Ultimately, owing to lower FPR and faster computational time, we suggest Horn’s parallel analysis as the default method for determining the number of PCs to retain in MI differential expression analysis with RNAseqCovarImpute.

Fig. S1: Overview of RNAseqCovarImpute MI Gene Bin Method


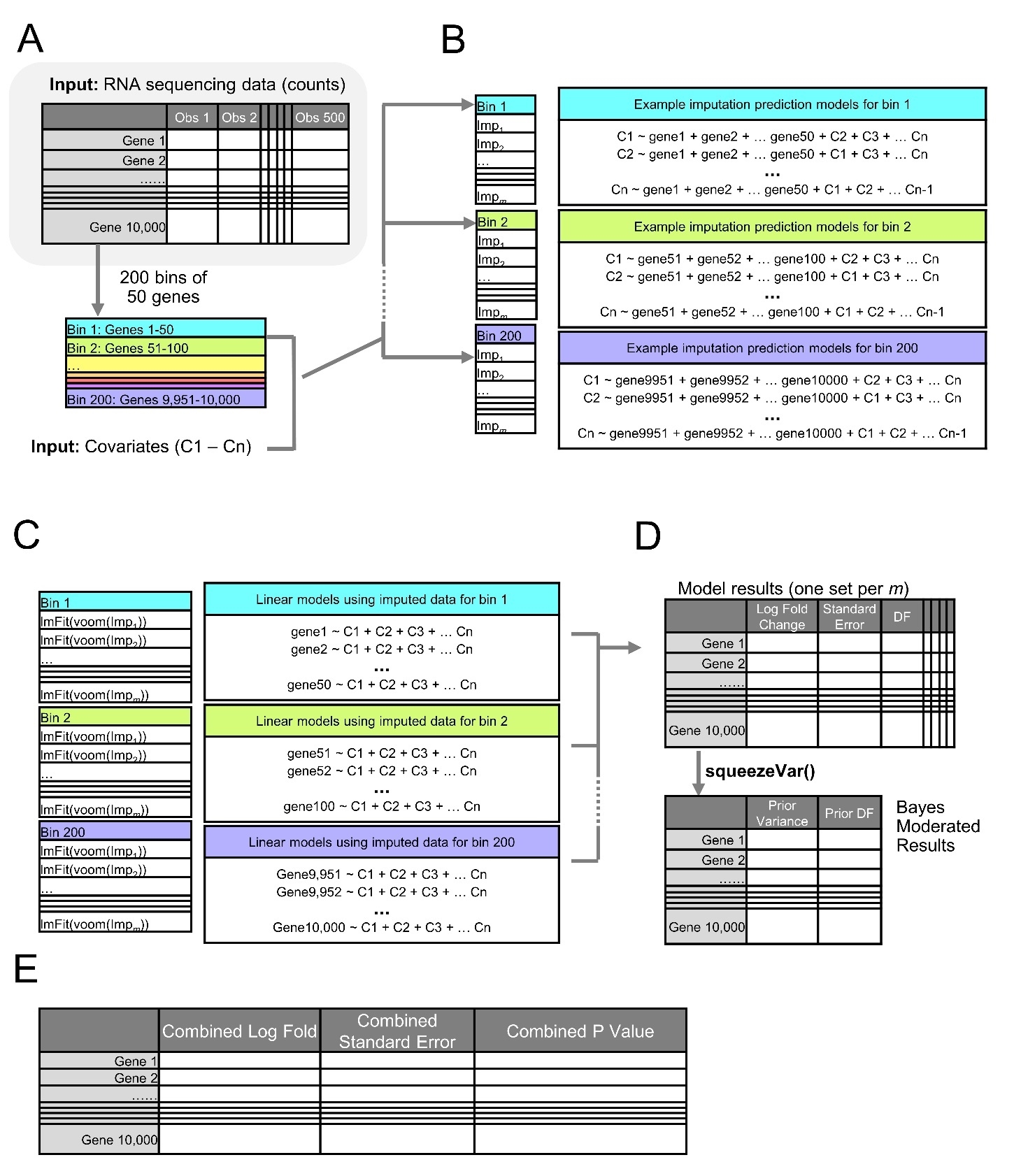


Legend: A) Starting with a matrix of counts for 10,000 genes across 500 observations, create 200 bins of 50 genes (default = 1 gene per 10 observations). B) Separately within each bin, create *m* imputed datasets using mice function. Along with covariates, the imputation predictor matrix includes the logCPM values for the genes within each bin. C) Estimate association of predictor of interest with gene expression using voom followed by lmFit functions. The lmFit linear models are run separately within each gene bin (each gene bin has its own set of imputed data created using those genes in the imputation predictor matrix). A modified voom function considers the entire library size of the starting count matrix. D) Un-bin genes and stack into *m* sets of model results. Use squeezeVar function separately on each *m* set of model results to apply empirical Bayes method to squeeze variances. E) Combine across *m* sets of model results using Rubin’s rules.

Fig. S2: Performance of RNAseqCovarImpute Methods on ECHO-PATHWAYS Dataset





Legend: (A) True positive rate (TPR), (B) false positive rate (FPR), and (C) mean absolute percentage error (MAPE) shown for RNAseqCovarImpute multiple imputation (MI) Gene Bin method, MI Principal Component Analysis (PCA) method retaining PCs explaining 80% of variation, and MI PCA method retaining PCs based on Horn’s parallel analysis. Differential expression analyses on ten datasets with simulated missingness per missingness mechanism per level of missingness. Box (median and interquartile range) and whiskers (1.5* interquartile range) shown along with one point per simulation. Dashed line at target FPR of 0.05.

Fig. S3: Performance of RNAseqCovarImpute Methods on NSCLC Dataset





Legend: (A) True positive rate (TPR), (B) false positive rate (FPR), and (C) mean absolute percentage error (MAPE) shown for RNAseqCovarImpute multiple imputation (MI) Gene Bin method, MI Principal Component Analysis (PCA) method retaining PCs explaining 80% of variation, and MI PCA method retaining PCs based on Horn’s parallel analysis. Differential expression analyses on ten datasets with simulated missingness per missingness mechanism per level of missingness. Box (median and interquartile range) and whiskers (1.5* interquartile range) shown along with one point per simulation. Dashed line at target FPR of 0.05.

Fig. S4: Performance of RNAseqCovarImpute Methods on EBV Dataset





Legend: (A) True positive rate (TPR), (B) false positive rate (FPR), and (C) mean absolute percentage error (MAPE) shown for RNAseqCovarImpute multiple imputation (MI) Gene Bin method, MI Principal Component Analysis (PCA) method retaining PCs explaining 80% of variation, and MI PCA method retaining PCs based on Horn’s parallel analysis. Differential expression analyses on ten datasets with simulated missingness per missingness mechanism per level of missingness. Box (median and interquartile range) and whiskers (1.5* interquartile range) shown along with one point per simulation. Dashed line at target FPR of 0.05.

Fig. S5: Coefficient Distributions of Synthetic RNA-sequencing Data


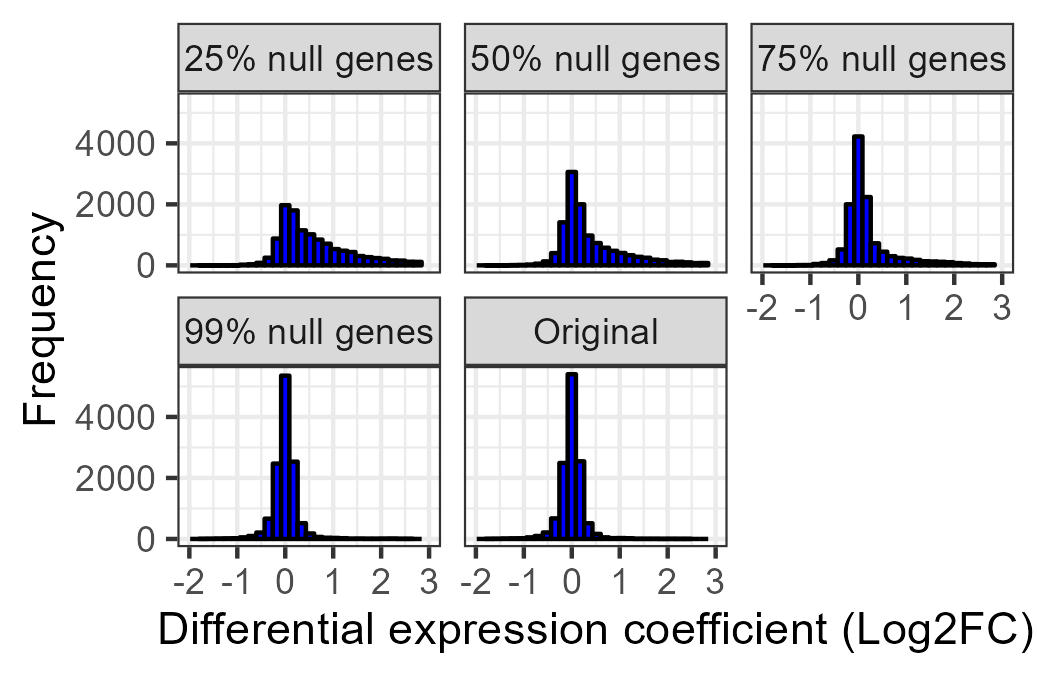


Legend: Desired coefficients (Log2 fold-changes) were input into the seqgendiff package to modify the NSCLC RNA-sequencing count matrix. Subsets of 25%-99% of genes were randomly selected to have their coefficient of association with sex set to zero. Coefficients for the remaining genes were drawn randomly from a gamma distribution.

Fig. S6: Synthetic RNA-sequencing Data Coefficients





Legend: Legend: Desired gene coefficients were input into the seqgendiff package to modify the NSCLC RNA-sequencing count matrix. Estimated coefficients come from the limma-voom pipeline applied to this modified count matrix in order to estimate the effect of sex on gene expression, controlling for participant age (continuous) and participant smoking status (smoker versus ex-smoker versus non-smoker). Data shown from four sets of synthetic RNA-sequencing data where the desired percent of null genes (differential expression coefficient for sex approximately = 0) was A) 25%, B) 50%, C) 75%, and D) 99%.

Fig. S7: True Positive Rate (TPR) Performance of Missing Data Methods on Synthetic RNA-sequencing Data





Legend: TPR shown for complete case (CC), single imputation (SI) and RNAseqCovarImpute (MI PCA Horn method) differential expression analyses on ten datasets with simulated missingness per missingness mechanism per level of missingness per synthetic dataset. Synthetic datasets had varying null gene rates.

Fig. S8: False Positive Rate (FPR) Performance of Missing Data Methods on Synthetic RNA-sequencing Data





Legend: FPR shown for complete case (CC), single imputation (SI) and RNAseqCovarImpute (MI PCA Horn method) differential expression analyses on ten datasets with simulated missingness per missingness mechanism per level of missingness per synthetic dataset. Synthetic datasets had varying null gene rates. Dashed line at target FPR of 0.05.

Fig. S9: RNAseqCovarImpute Computation Time


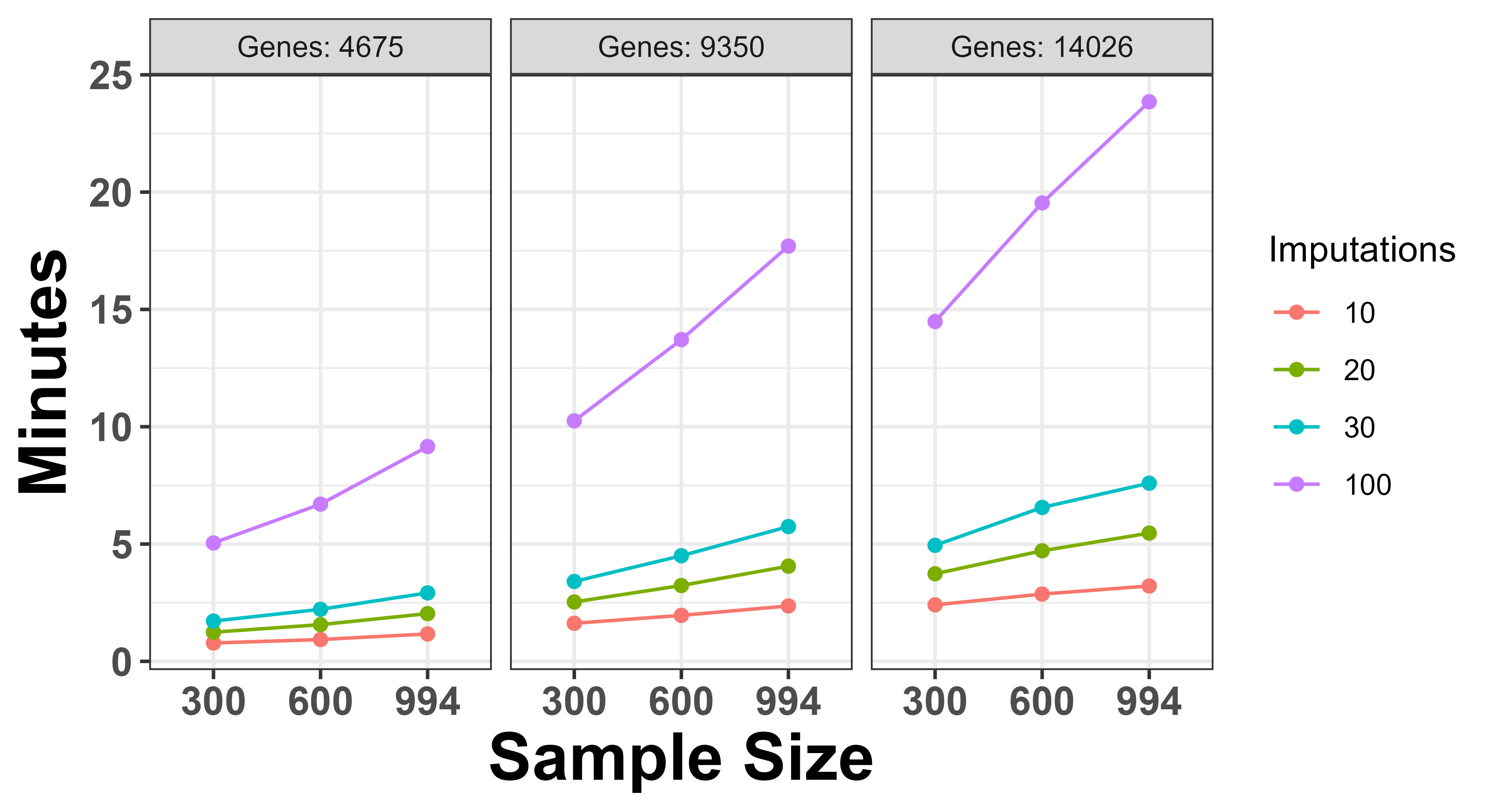


Legend: RNAseqCovarImpute MI PCA method was benchmarked in an analysis of the ECHO-PATHWAYS dataset with 14,026 genes, 4 covariates in the model, and 55% missingness under MCAR on a Windows machine with 3.8 GHz processing speed and 16GB random-access memory. Analyses were run in parallel with up to 14 workers. Mean run time shown over three iterations per combination of sample size, number of genes, and number of imputed datasets.

Fig. S10: Overlapping DEGs from Maternal Age and the Placental Transcriptome Differential Expression Analysis


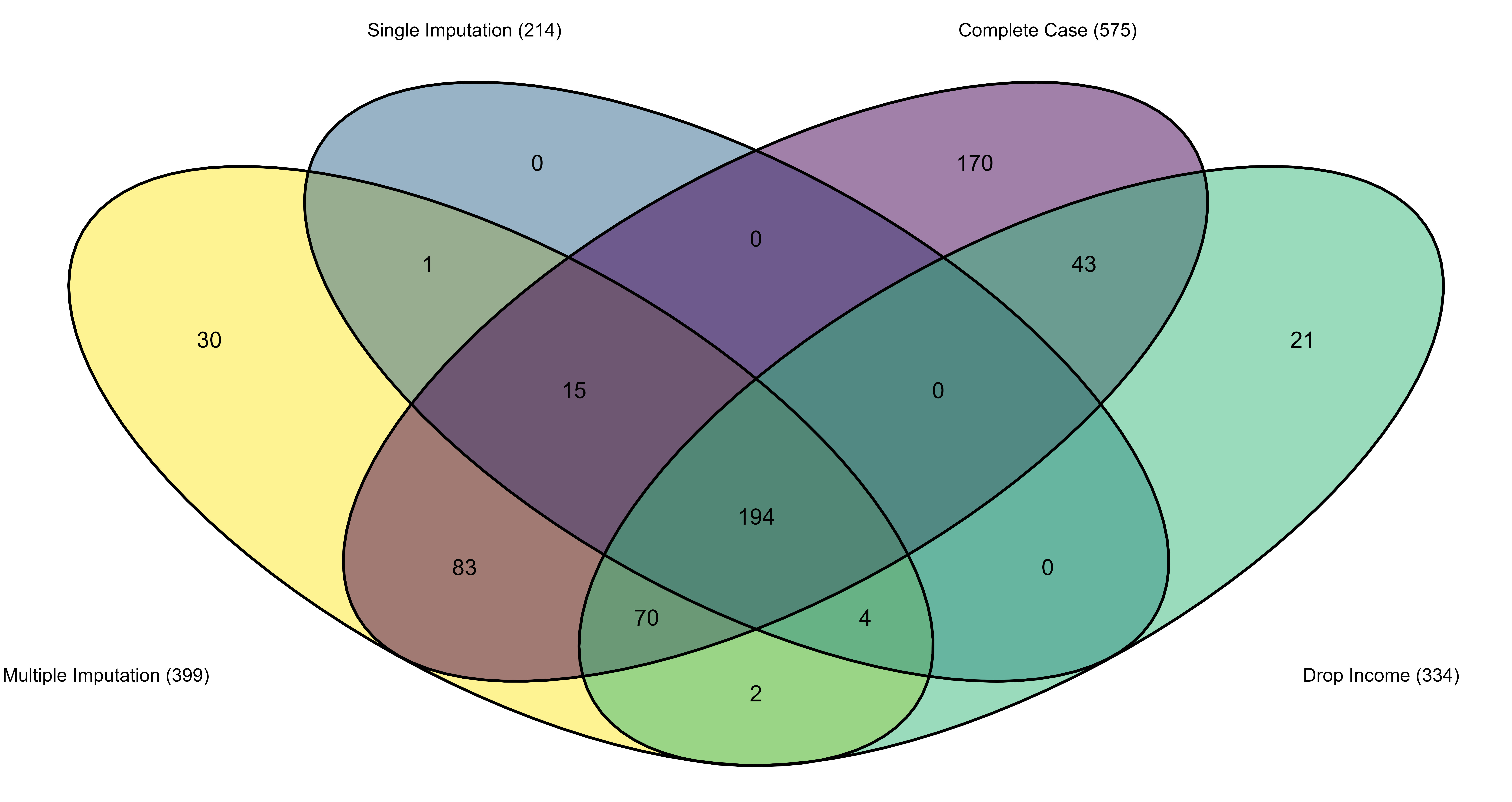


Legend: Venn diagram depicts shared and distinct differentially expressed genes (DEGs) for each method. “Drop Income” indicates complete case analysis excluding the income covariate.

Fig. S11: Imputation Diagnostics


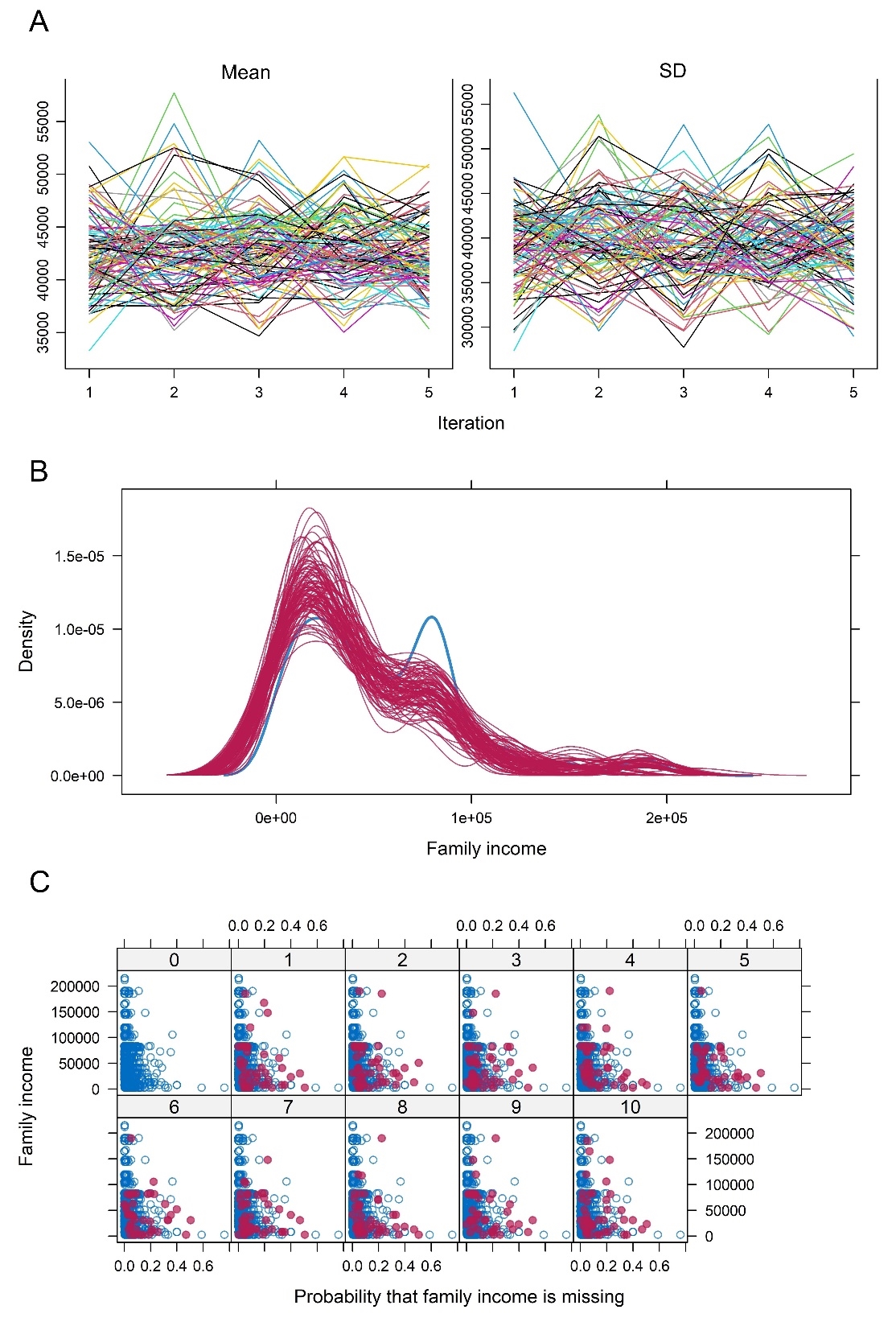


Legend: Imputation diagnostics for family income variable for the analysis of maternal age and the placental transcriptome. (A) trace plot shows that lines converge rather than deviating towards individual trends. (B) density plot and (C) scatter plot for the first 10 imputed datasets shows that distributions of the observed (blue) and imputed (red) data are similar.

Fig. S12: Colorectal Carcinoma and the Blood Platelet Transcriptome Differential Expression Analysis





Legend: Venn diagram depicts shared and distinct differentially expressed genes for each method (A). P-value rankings for each method for the top 10 genes with the lowest P-values from the multiple imputation analysis (B). Volcano plots of differential gene expression in blood platelets from individuals with colorectal carcinoma versus healthy donors in complete case (C), single imputation (D) and multiple imputation (E) analyses. Models include the following covariates: cancer status, genotype, age, sex, and sequencing batch. Horizontal and vertical lines at P = 0.05 and Log_2_-adjusted fold-changes (LogFC) = -6.0, respectively. HGNC gene symbols shown for significant genes with false discovery rate adjusted P-value (P-adj) <0.05 and LogFC beyond -6.0 cutoff.
