# Supplementary material for: RNAseqCovarImpute: a multiple imputation procedure that outperforms complete case and single imputation differential expression analysis
Source: Genome Biol. 2024 Sep 3;25:236. doi: 10.1186/s13059-024-03376-7 (PMC11370143; doi:10.1186/s13059-024-03376-7)
Supplement: Supplementary file 2 — Additional file 2. The review history. [file 13059_2024_3376_MOESM2_ESM.docx]

**1^st^ round**

**Reviewer 1**

The manuscript entitled "RNAseqCovarImpute: a multiple imputation procedure that outperforms complete case and single imputation differential expression analysis" by Baker and colleagues describes a procedure for imputing covariates for differential expression analysis of RNA-seq data using multiple imputation. The authors relied heavily on existing packages to implement the RNAseqCovarImpute package, using the mice package for multiple imputation and limma-voom for differential expression analysis. The authors used missing completely at random (MCAR) and missing at random (MAR) examples to perform simulations to show that the proposed method reduces bias while maintaining power.

There are several aspects in which the manuscript can be significantly improved.

First, the authors described the method as a "tool", but did not comprehensively describe their "method". In particular, any mathematical details of how multiple imputation was performed or how differential expression was performed are completely missing, and the reader has to read other relevant papers to figure this out. This made the manuscript extremely difficult to read or review. The manuscript significantly loses the interest of statistically oriented readers. A mathematical description of how the method works must be included in the revised manuscript. The parameter settings should be explained not only with respect to the tools, but also with respect to the mathematical models.

Second, the plots are poorly organized and presented. In Figure 1, the authors made no attempt to distinguish what was a predictor variable, a covariate, or a gene expression value. The figure did not really help to understand the overall architecture of the proposed methods and tools. It was unclear why the input included only RNA-seq data, but not covariates. In Figure 2-4, the labeling of the y-axis was uninformative or misleading. The legends suggest that the top numbers represent errors, but I doubt that the description is correct.

Third, the simulation settings were not described well enough to reproduce the results. There is not enough description of the criteria for deciding "true positives" vs. "false positives" when using real data. When simulating synthetic data, the effect size distribution was not clearly described. Even when some details were described, the information was scattered, making it very difficult for readers to understand the overall picture of the simulation settings. A mathematical description of the simulation settings is highly recommended.

Fourth, the authors only simulated the missingness with MCAR and MAR scenario, so it is unclear how robust the proposed method will be when there is systematic correlation between the patterns of missingness, which is common in real dataset. It is important to understand in which scenario of missingness patterns the proposed method would work and when it would not. It would be hard to imagine that multiple imputation methods assuming random missingness would outperform full case-control analysis in terms of power and false positives in all scenarios.

In addition, some of the links provided in the manuscript were not accessible, making it difficult to independently test the tool.

**Reviewer 2**

Leveraging the benefits of multiple imputation (MI) method, the authors propose a comprehensive procedure, RNAseqCovarImpute, that concurrently imputes covariates within the transcriptomic dataset. Utilizing the imputed covariates and transcriptomic data, the limma-voom algorithm and Rubin's rules are employed for the identification of Differentially Expressed Genes (DEGs) associated with the covariates. By multiple well-designed simulation experiments, the authors show the superiority of RNAseqCovarImpute in term of identifying true positives, controlling False Discovery Rate (FDR) and limiting bias of DEGs compared with complete case and single imputation differential expression analysis. By applying the RNAseqCovarImpute on the real RNA-seq data from ECHO-PATHWAYS, the authors delineate the placental transcriptomic changes associated with maternal age. Although the authors provide a reasonable method for predicting the missing covariates in data, there exists some questions that the authors should consider:

(1) The authors emphasize the imputation of missing covariates in RNA-seq data as the central motivation behind RNAseqCovarImpute. However, Figure 1 solely illustrates the process of RNAseqCovarImpute for DEGs in RNA-seq data and does not elucidate the imputation process. Clarity would be enhanced if the authors could depict the missingness in RNA-seq data and demonstrate how RNAseqCovarImpute facilitates the imputation of these missing covariates.

(2) Being an R package, RNAseqCovarimpute lacks a clear explanation of its input and output in the "Multiple imputation and differential expression analysis in RNAseqCovarimpute package" section on page 6. Clarity could be greatly improved if the authors define the input and output using mathematical notation, making it more accessible for users to understand the functioning of the package.

(3) RNAseqCovarImpute provides a method that integrates MI and limma-voom to identify Differentially Expressed Genes (DEGs) associated with interesting covariates. Although limma is a widely used tool for differential expression analysis, the enhanced version, limma-voom, specifically designed to address heteroscedasticity in RNA-seq data, may be less familiar to readers without a strong statistical background. Therefore, introducing a series of mathematical notations to illustrate the gene binning process, the generation of M imputed datasets for fitting the LOWESS curve to obtain an optimal fit, and the application of Rubin's rules for aggregating multiple results would enhance the accuracy and rigor of the article.

(4) The RNAseqCovarImpute is broadly divided into two components: covariate imputation using mice and differential expression analysis employing limma-voom. In the simulation experiments, the authors establish the efficiency of RNAseqCovarImpute in identifying Differentially Expressed Genes (DEGs) based on imputed data, comparing favorably with complete case and single imputation methods. However, the authors do not thoroughly investigate the efficacy of the imputation module of RNAseqCovarImpute. Referring to [1], numerous methods for imputing clinical data exist, with mice not necessarily demonstrating superior performance compared to other methods. Therefore, it is suggested that the authors design additional simulation scenarios to specifically demonstrate the advantages of mice in comparison to other imputation methods when applied to RNA-seq data.

(5) Based on the tutorials provided in the GitHub repository, RNAseqCovarImpute appears proficient in imputing gene expression in RNA-seq data. However, there are numerous imputation methods designed for single-cell transcriptome data, such as MAGIC [2], SAVER [3], and scVI [4]. Therefore, it is recommended that the authors undertake a comparative analysis of RNAseqCovarImpute against these methods. This analysis should specifically focus on given covariates within simulated RNA-seq data, offering valuable insights into the imputation accuracy achieved by RNAseqCovarImpute.

(6) In the section "Application of RNAseqCovarImpute in analysis of maternal age and placental transcriptome," it is recommended that the authors introduce pathway analysis or gene set enrichment analysis. This addition would offer further evidence supporting the notion that the identified DEGs play crucial biological roles in spontaneous preterm birth.

(7) In the section "Application of RNAseqCovarImpute in analysis of maternal age and placental transcriptome," it is recommended that the authors provide a more comprehensive analysis. This should include an exploration of transcriptomic differences among various groups, such as those categorized by delivery method, maternal alcohol consumption during pregnancy, maternal tobacco using during pregnancy, and other relevant factors. Expanding the analysis to consider these variables would contribute to a more thorough understanding of the transcriptomic landscape in relation to maternal age and its interactions with various conditions.

(8) The authors are encouraged to extend the application of RNAseqCovarImpute to a broader range of RNA-seq datasets, especially those containing clinical data with missing covariates. Examples could include cancer data from the Cancer Genome Atlas Program (TCGA). This expansion of the application would further validate the utility and robustness of RNAseqCovarImpute across diverse datasets and enhance its generalizability to different biological contexts.

(9) To provide emphasis on the highlights of RNAseqCovarImpute, the authors are recommended to rephrase the contents in the fourth paragraph of the Background section.

(10) In Figures 2-7, it appears that the contents become blurred when zoomed in. The authors are advised to carefully review the images and consider using vector graphics within the article to ensure optimal clarity and resolution, especially upon closer examination.

(11) In Figure 2-4(A) and Figure 5(A), the utilization of "%" on the y-axis might be confusing for readers. It is suggested that the authors consider dividing the figure into two parts and explicitly label each section of the y-axis (FDR and true positives). Additionally, it would be beneficial to include a black line indicating the target False Positive Rate (FPR) of 5% for enhanced clarity and reference [5].

(12) The contents in the second paragraph of the Discussion (Page 11) appear redundant with the information presented in the second paragraph of the Background (Page 4). It is advised to revise and streamline these sections to avoid repetition and ensure a more concise and focused presentation of the key points.

References:

[1] Luo Y. Evaluating the state of the art in missing data imputation for clinical data[J]. Briefings in Bioinformatics, 2022, 23(1): bbab489.

[2] Van Dijk D, Sharma R, Nainys J, et al. Recovering gene interactions from single-cell data using data diffusion[J]. Cell, 2018, 174(3): 716-729. e27.

[3] Huang M, Wang J, Torre E, et al. SAVER: gene expression recovery for single-cell RNA sequencing[J]. Nature methods, 2018, 15(7): 539-542.

[4] Lopez R, Regier J, Cole M B, et al. Deep generative modeling for single-cell transcriptomics[J]. Nature methods, 2018, 15(12): 1053-1058.

[5] Soneson C, Robinson M D. Bias, robustness and scalability in single-cell differential expression analysis[J]. Nature methods, 2018, 15(4): 255-261.

**Authors’ response**

Following reviewer comments, we tested a new covariate imputation method not presented in the original article. The new method is also a version of multiple imputation (MI), in the spirit of the original aim to improve bulk RNA-sequencing differential expression analysis in large studies with missing data by applying MI. To our knowledge, all other differential expression analysis studies in large observational settings with missing covariate data have used either complete case analysis, where any participant with at least one missing covariate is dropped, or single imputation with random forest.

The main issue with using MI in transcriptomic studies is that the outcome must be included in the imputation prediction model, a requirement of MI to avoid bias. Our original MI method overcame the difficulty of including such high dimensional RNA-sequencing data in the predictive model by binning genes into smaller groups (i.e., 50-200 depending on sample size). All ~10,000-20,000 genes in an analysis cannot be reasonably included in a multiple imputation prediction model, but a smaller subset of 50-200 genes can be included.

Looping through these gene bins and conducting MI and differential expression analysis separately within each bin is computationally intensive. Additionally, each instance of MI only has information on a small subset of genes, and information from the rest of the transcriptome is lost.

The new method we developed uses principal component analysis (PCA) to overcome these issues while still satisfying the requirement that outcome information is included in the MI predictive models. We conduct PCA for the entire transcriptome, and include the top PCs in the imputation predictive modeling to make *m* imputed datasets. In this MI PCA method, we only need to run a single set of MI and differential expression analysis, rather than needing to rerun the analysis for each bin of genes in the MI Gene Bin method. The MI PCA method is about 20 times faster, depending on the number of input genes and sample size. Additionally, the MI predictive modeling benefits from having information on the entire transcriptome (in the form of the top PCs from PCA) rather than information on just a subset of genes. We compared the MI PCA method to the MI Gene Bin method in the supplement and found that the MI PCA method had slightly better performance. In the revised article, we now describe the MI PCA method as the main method of choice in our R package, and compare it with complete case and single imputation in the main text.

Reviewer #1

The manuscript entitled "RNAseqCovarImpute: a multiple imputation procedure that outperforms complete case and single imputation differential expression analysis" by Baker and colleagues describes a procedure for imputing covariates for differential expression analysis of RNA-seq data using multiple imputation. The authors relied heavily on existing packages to implement the RNAseqCovarImpute package, using the mice package for multiple imputation and limma-voom for differential expression analysis. The authors used missing completely at random (MCAR) and missing at random (MAR) examples to perform simulations to show that the proposed method reduces bias while maintaining power.

There are several aspects in which the manuscript can be significantly improved.

First, the authors described the method as a "tool", but did not comprehensively describe their "method". In particular, any mathematical details of how multiple imputation was performed or how differential expression was performed are completely missing, and the reader has to read other relevant papers to figure this out. This made the manuscript extremely difficult to read or review. The manuscript significantly loses the interest of statistically oriented readers. A mathematical description of how the method works must be included in the revised manuscript. The parameter settings should be explained not only with respect to the tools, but also with respect to the mathematical models.

We have added mathematical models to describe the methods in more detail, thank you for the suggestion. See Methods lines 260-378.

Second, the plots are poorly organized and presented. In Figure 1, the authors made no attempt to distinguish what was a predictor variable, a covariate, or a gene expression value. The figure did not really help to understand the overall architecture of the proposed methods and tools. It was unclear why the input included only RNA-seq data, but not covariates.

We have updated this plot to more clearly define the covariates and the genes as the inputs. The figure now also shows how these variables are used in the imputation process, and in the differential expression analysis. This appears in Supplemental Figure 1. The new Figure 1 describes the MI PCA method, and we also made sure to clearly show gene expression and covariate data as the input.

In Figure 2-4, the labeling of the y-axis was uninformative or misleading. The legends suggest that the top numbers represent errors, but I doubt that the description is correct.

Thanks for pointing this out, we realized that our format for reporting performance was not conventional. We renamed “True positives” to true positive rate (TPR), which is defined as the proportion of actual DEGs from the full data models that were correctly identified as DEGs following simulated missingness, and we renamed what was previously FDR to the false positive rate (FPR) to be consistent. FPR was the proportion of false positives out of the total number of significant results. Additionally, our older metric of “bias” reflected differences in true coefficients for genes and the coefficients uncovered after simulated missingness and imputation (or complete case (CC) analysis), but this metric was not conventional. We now instead report the mean absolute percentage error (MAPE) across all true DEGs to characterize the ability of each method to reproduce gene expression coefficients from the full data model. See the “Evaluating results” section of the methods starting at line 418 for details.

We also updated the performance plots to show boxplots and individual points rather than mean +- SEM.

Third, the simulation settings were not described well enough to reproduce the results. There is not enough description of the criteria for deciding "true positives" vs. "false positives" when using real data. When simulating synthetic data, the effect size distribution was not clearly described. Even when some details were described, the information was scattered, making it very difficult for readers to understand the overall picture of the simulation settings. A mathematical description of the simulation settings is highly recommended.

We have removed the synthetic RNAseq data and added in two more real datasets (also following suggestions by the other reviewer to utilize more real data). For real datasets, the definition of true positive is more clear: genes significantly differentially expressed in the full dataset (before missingness is simulated). The goal of imputing missing covariate data is to recover the same model results that we would have gotten if we had all of the covariate data with no missingness to begin with. Genes erroneously identified as significant by a given method (MI, SI, or CC) that were not truly differentially expressed based on the full data analysis were defined as false positives. This is described in the methods under “Evaluating results”.

Fourth, the authors only simulated the missingness with MCAR and MAR scenario, so it is unclear how robust the proposed method will be when there is systematic correlation between the patterns of missingness, which is common in real dataset. It is important to understand in which scenario of missingness patterns the proposed method would work and when it would not. It would be hard to imagine that multiple imputation methods assuming random missingness would outperform full case-control analysis in terms of power and false positives in all scenarios.

Thank you for this suggestion – we now also include MNAR. See the “Simulating missing data under different missingness mechanisms: section of the methods starting at line 393.

To clarify, data under the MAR missing mechanism also have systematic correlation between the patterns of missingness. The difference between MAR and MNAR is that in MAR, the data that contribute to these patterns are available, while in MNAR, the data (or some of the data) responsible for the patterns of missingness are not available (see lines 404-411 in our revision). It could be that these variables contributing to the missingness of other covariates were not measured, or that we don’t know which variables contribute to the missingness so we fail to include them. Another point of clarification is that we never show (or expected) that multiple imputation, single imputation, or complete case analysis would outperform the full data analysis (our examples include both case-control (i.e., male versus female) and continuous predictors as the variables of interest). The full data analysis is the baseline truth that we hope these mechanisms for handling missing data can recreate. 

In addition, some of the links provided in the manuscript were not accessible, making it difficult to independently test the tool.
Thank you for bringing this to our attention. Since our initial submission, the package has been added to the Bioconductor repository. Through this transition we may have not been careful to ensure the links provided at the time of submission remained active, and we apologize for this inconvenience. In the resubmission, we provide links to the package on Bioconductor, and links to GitHub with code specific to this analysis. Here are those links for convenience:

“The RNAseqCovarImpute package is available at the Bioconductor repository (<https://bioconductor.org/packages/release/bioc/html/RNAseqCovarImpute.html>). Code for simulating missing covariate data and evaluating different methods for handling missing data, including the complete case, single imputation, and RNAseqCovarImpute methods is available on GitHub (<https://github.com/brennanhilton/RNAseqCovarImpute_NSCLC_data>).”

Reviewer #2

Leveraging the benefits of multiple imputation (MI) method, the authors propose a comprehensive procedure, RNAseqCovarImpute, that concurrently imputes covariates within the transcriptomic dataset. Utilizing the imputed covariates and transcriptomic data, the limma-voom algorithm and Rubin's rules are employed for the identification of Differentially Expressed Genes (DEGs) associated with the covariates. By multiple well-designed simulation experiments, the authors show the superiority of RNAseqCovarImpute in term of identifying true positives, controlling False Discovery Rate (FDR) and limiting bias of DEGs compared with complete case and single imputation differential expression analysis. By applying the RNAseqCovarImpute on the real RNA-seq data from ECHO-PATHWAYS, the authors delineate the placental transcriptomic changes associated with maternal age. Although the authors provide a reasonable method for predicting the missing covariates in data, there exists
some questions that the authors should consider:

(1) The authors emphasize the imputation of missing covariates in RNA-seq data as the central motivation behind RNAseqCovarImpute. However, Figure 1 solely illustrates the process of RNAseqCovarImpute for DEGs in RNA-seq data and does not elucidate the imputation process. Clarity would be enhanced if the authors could depict the missingness in RNA-seq data and demonstrate how RNAseqCovarImpute facilitates the imputation of these missing covariates.

To clarify, information related to RNA transcripts or RNA-seq data are not imputed in this method.  The missing data would solely exist for covariate data and not for sequencing data. The multiple imputation procedure is for covariates using the “MICE” method. In this paper, we rely on well-established multiple imputation approaches that are used broadly in the epidemiology field (1-3). The gap we aim to fill is that multiple imputation is considered a gold standard, especially in epidemiology, but it is not compatible with high-dimensional outcome data like in RNA-sequencing or other omics. We now clarify this better in the fourth paragraph of the introduction –

Line 46: “To ensure that outcome data are included in the predictive model (a requirement of MI to avoid bias (7)), one solution is to …”

And Line 53:

“We propose an alternative solution for applying MI to high dimensional gene expression data, which is to utilize principal component analysis (PCA) to reduce the dimensionality of the transcriptome. Then, the top PCs can be included in the MI prediction model when imputing missing covariates, satisfying the requirement that outcome information is included in the MI predictive models.”

(2) Being an R package, RNAseqCovarimpute lacks a clear explanation of its input and output in the "Multiple imputation and differential expression analysis in RNAseqCovarimpute package" section on page 6. Clarity could be greatly improved if the authors define the input and output using mathematical notation, making it more accessible for users to understand the functioning of the package.
We have added mathematical models to describe the methods in more detail, thank you for the suggestion. See Methods lines 260-378. We also updated the figures describing the method (Figure 1 and Supplemental Figure 1). These now better demonstrate the input RNA-seq and input covariate data, how these data are utilized in the covariate imputation process, and how they are utilized in the differential expression analysis. Additionally, the R package is now available on Bioconductor (<https://bioconductor.org/packages/release/bioc/html/RNAseqCovarImpute.html>). This R package now includes a detailed vignette that outlines the inputs (matrix or DGE list of RNA-seq counts and data frame of covariates) and outputs (logFC coefficients, standard errors, and p-values).

(3) RNAseqCovarImpute provides a method that integrates MI and limma-voom to identify Differentially Expressed Genes (DEGs) associated with interesting covariates. Although limma is a widely used tool for differential expression analysis, the enhanced version, limma-voom, specifically designed to address heteroscedasticity in RNA-seq data, may be less familiar to readers without a strong statistical background. Therefore, introducing a series of mathematical notations to illustrate the gene binning process, the generation of M imputed datasets for fitting the LOWESS curve to obtain an optimal fit, and the application of Rubin's rules for aggregating multiple results would enhance the accuracy and rigor of the article.

We have added mathematical models to describe the methods in more detail, thank you for the suggestion. See Methods lines 260-378.

(4) The RNAseqCovarImpute is broadly divided into two components: covariate imputation using mice and differential expression analysis employing limma-voom. In the simulation experiments, the authors establish the efficiency of RNAseqCovarImpute in identifying Differentially Expressed Genes (DEGs) based on imputed data, comparing favorably with complete case and single imputation methods. However, the authors do not thoroughly investigate the efficacy of the imputation module of RNAseqCovarImpute. Referring to [1], numerous methods for imputing clinical data exist, with mice not necessarily demonstrating superior performance compared to other methods. Therefore, it is suggested that the authors design additional simulation scenarios to specifically demonstrate the advantages of mice in comparison to other imputation methods when applied to RNA-seq data.

We thank the reviewer for this suggestion. We did try out two new methods, the MI PCA method that is now the default method in our R package, and a machine learning method newly developed with a corresponding R package (MLIM: <https://cran.r-project.org/web/packages/mlim/readme/README.html>). We found that MLIM performed about the same as the random forest SI method that we present currently (see Figure 1 below), and elected to remove this in the final text.

To the best of our knowledge, nearly all large observational studies of the transcriptome with multivariate statistical models including variables with missing data have utilized complete case analysis (4-6), while very few have instead employed single imputation with random forest (7). The majority of RNA sequencing studies have complete covariate/clinical data available, so it makes sense that there has been little effort to determine the best way to handle missing covariate data in this context. MICE is considered the gold standard in human observational studies for handling missing data, but has not been utilized in transcriptomic studies. This was our reasoning for testing MICE against complete case analysis and single imputation with random forest.

We agree that alternative imputation methods should be tested in the context of RNA-sequencing, but believe that testing such alternative methods in depth should be the subject of future work, once these methods (including some promising emerging machine learning methods) gain more popularity and validation. We have added the following sentence with reference to [1] to the discussion to reflect this, lines 244-246:

 “Finally, data imputation is a rapidly evolving field, and emerging machine learning SI and MI methods (32) should be tested in the context of RNA-sequencing in future studies.”

Here is the figure (Figure 1) showing the new method (MLIM, green) performed about the same as SI with random forest (purple), and that the MI PCA Horn method in RNAseqCovarImpute is the best performer.

Figure 1

(5) Based on the tutorials provided in the GitHub repository, RNAseqCovarImpute appears proficient in imputing gene expression in RNA-seq data. However, there are numerous imputation methods designed for single-cell transcriptome data, such as MAGIC [2], SAVER [3], and scVI [4]. Therefore, it is recommended that the authors undertake a comparative analysis of RNAseqCovarImpute against these methods. This analysis should specifically focus on given covariates within simulated RNA-seq data, offering valuable insights into the imputation accuracy achieved by RNAseqCovarImpute.

We are unable to compare RNAseqCovarImpute to these methods, as we do not impute missing RNA-seq data, only missing covariate data, and believe that those methods fill different roles compared with the RNAseqCovarImpute method.

(6) In the section "Application of RNAseqCovarImpute in analysis of maternal age and placental transcriptome," it is recommended that the authors introduce pathway analysis or gene set enrichment analysis. This addition would offer further evidence supporting the notion that the identified DEGs play crucial biological roles in spontaneous preterm birth.

Thank you for this suggestion. We now additionally include enrichment analysis of KEGG pathways associated with maternal age. This is described in the results lines 166-171:

“Pathway analysis revealed 32 Kyoto Encyclopedia of Genes and Genomes (KEGG) pathways that were downregulated in association with older maternal age (Figure 7). Among these downregulated KEGG pathways, 11 belong to the immune system KEGG group and 6 belong to the signal transduction group.  Antigen processing and presentation, an immune system KEGG pathway, was the most strongly downregulated pathway according to its enrichment effect size and P-value (Figure 7).”

We did find that many of the signals uncovered here were also linked with preterm birth in a prior analysis. This is described in the discussion lines 226-231:

“Advanced maternal age is a well-known risk factor for preterm birth (28, 29). These analyses demonstrated associations of advanced maternal age with downregulation of individual genes (i.e., CXCL8) and pathways (i.e., 9 immune system and 4 signal transduction pathways) that were also downregulated in association with spontaneous preterm birth in a prior analysis (30). Future studies should formally explore these overlapping results as putative mechanistic links between advanced maternal age and preterm birth.”

(7) In the section "Application of RNAseqCovarImpute in analysis of maternal age and placental transcriptome," it is recommended that the authors provide a more comprehensive analysis. This should include an exploration of transcriptomic differences among various groups, such as those categorized by delivery method, maternal alcohol consumption during pregnancy, maternal tobacco using during pregnancy, and other relevant factors. Expanding the analysis to consider these variables would contribute to a more thorough understanding of the transcriptomic landscape in relation to maternal age and its interactions with various conditions.

The goal for this manuscript was to describe and test this statistical method for missing covariate data in RNA sequencing studies, with maternal age used as an example. These data (the ECHO-PATHWAYS data) are the subject of many completed and ongoing placental transcriptomic analyses. Prior work has looked at the placental transcriptome in these data in association with spontaneous preterm birth (<https://www.ncbi.nlm.nih.gov/pmc/articles/PMC9790028/> ), maternal stress (<https://doi.org/10.1038/s41380-023-02403-6> ), exposure to particulate matter (<https://doi.org/10.1016/j.envint.2022.107310> ) and exposure to phthalates (<https://doi.org/10.1289/EHP8973> ). Ongoing work that is currently in preparation or under review in other manuscripts examines other variables including nutrition factors, pharmaceutical/drug use, birthweight, gestational age, delivery method, and additional environmental exposures. Our method will be used as part of these analyses to impute missing covariate data to improve sample size and subsequent statistical power.  Although we believe this analysis adds valuable data on maternal age and placental gene expression, we intended it to be a proof-of-concept for this new MI method. We also do not want to take away from future analyses that may focus on these other factors more in depth.

(8) The authors are encouraged to extend the application of RNAseqCovarImpute to a broader range of RNA-seq datasets, especially those containing clinical data with missing covariates. Examples could include cancer data from the Cancer Genome Atlas Program (TCGA). This expansion of the application would further validate the utility and robustness of RNAseqCovarImpute across diverse datasets and enhance its generalizability to different biological contexts.

Thank you for this suggestion, we now include two additional real RNA-sequencing datasets that are publicly available from the European Molecular Biology Laboratory - European Bioinformatics Institute (EMBL-EBI) repository. We only chose this repository over the TCGA because the data are readily available to download, while the TCGA has a data request process whereby only qualified, independent researchers can access the data.

<https://www.ebi.ac.uk/gxa/experiments/E-GEOD-81089/Results>

<https://www.ebi.ac.uk/gxa/experiments/E-MTAB-7805/Results>

In the text we call these the non-small cell lung cancer (NSCLC) dataset and the Epstein-Barr virus (EBV) dataset (see the “Datasets” section of the methods starting at line 433). In our experience many RNA-seq studies only include one variable and are matched on other factors so that other covariates do not need to be included in the models. The NSCLC and EBV datasets were the largest sample size RNA-seq datasets that we could find with at least 3 covariates.

(9) To provide emphasis on the highlights of RNAseqCovarImpute, the authors are recommended to rephrase the contents in the fourth paragraph of the Background section.

We now better emphasize that our method ensures that outcome data are included in the multiple imputation predictive model, which is a requirement of MI to avoid bias (Lines 46-57).

(10) In Figures 2-7, it appears that the contents become blurred when zoomed in. The authors are advised to carefully review the images and consider using vector graphics within the article to ensure optimal clarity and resolution, especially upon closer examination.

We appreciate that you have brought this to our attention. We are providing dpi 600 figures in the revision. If accepted, we will be sure to upload high quality images on their own (rather than in a single PDF with the article text).

(11) In Figure 2-4(A) and Figure 5(A), the utilization of "%" on the y-axis might be confusing for readers. It is suggested that the authors consider dividing the figure into two parts and explicitly label each section of the y-axis (FDR and true positives). Additionally, it would be beneficial to include a black line indicating the target False Positive Rate (FPR) of 5% for enhanced clarity and reference [5].

Thank you for pointing out that the axes were not clear. We renamed “True positives” to true positive rate (TPR), which is defined as the proportion of actual DEGs from the full data models that were correctly identified as DEGs following simulated missingness, and we renamed what was previously FDR as the false positive rate (FPR) to be consistent. FPR was the proportion of false positives out of the total number of significant results. We added a line at 0.05 as the target false discovery rate. We also now report the mean absolute percentage error (MAPE) as a more conventional measure of coefficient similarity between the true model and the missing data models. See the “Evaluating results” section of the methods starting at line 418 for details.
(12) The contents in the second paragraph of the Discussion (Page 11) appear redundant with the information presented in the second paragraph of the Background (Page 4). It is advised to revise and streamline these sections to avoid repetition and ensure a more concise and focused presentation of the key points.

We removed the redundant information, thanks! This portion was taken out of the discussion:

“Lack of development in this area may reflect the fact that a large proportion of RNA-sequencing studies are conducted in in-vitro or in vivo models and do not suffer from missing covariate data. Complete datasets are common in experimental studies with controlled conditions and a limited number of covariates. In an experimental setting, studies may employ two-group analyses with no additional variables or utilize covariates for which collecting data is trivial (e.g., sequencing batch and sex). Missing covariate data is ubiquitous, however, in large human observational studies.”

References:
[1] Luo Y. Evaluating the state of the art in missing data imputation for clinical data[J]. Briefings in Bioinformatics, 2022, 23(1): bbab489.
[2] Van Dijk D, Sharma R, Nainys J, et al. Recovering gene interactions from single-cell data using data diffusion[J]. Cell, 2018, 174(3): 716-729. e27.
[3] Huang M, Wang J, Torre E, et al. SAVER: gene expression recovery for single-cell RNA sequencing[J]. Nature methods, 2018, 15(7): 539-542.
[4] Lopez R, Regier J, Cole M B, et al. Deep generative modeling for single-cell transcriptomics[J]. Nature methods, 2018, 15(12): 1053-1058.
[5] Soneson C, Robinson M D. Bias, robustness and scalability in single-cell differential expression analysis[J]. Nature methods, 2018, 15(4): 255-261.

References for the author response

1.	Van Buuren S. Flexible imputation of missing data: CRC press; 2018.

2.	Mackinnon A. The use and reporting of multiple imputation in medical research–a review. Journal of internal medicine. 2010;268(6):586-93.

3.	Hayati Rezvan P, Lee KJ, Simpson JA. The rise of multiple imputation: a review of the reporting and implementation of the method in medical research. BMC medical research methodology. 2015;15:1-14.

4.	Enquobahrie DA, MacDonald J, Hussey M, Bammler TK, Loftus CT, Paquette AG, et al. Prenatal exposure to particulate matter and placental gene expression. Environment international. 2022;165:107310.

5.	Paquette AG, MacDonald J, Lapehn S, Bammler T, Kruger L, Day DB, et al. A comprehensive assessment of associations between prenatal phthalate exposure and the placental transcriptomic landscape. Environmental health perspectives. 2021;129(9):097003.

6.	Paquette AG, Lapehn S, Freije S, MacDonald J, Bammler T, Day DB, et al. Placental Transcriptomic Signatures of Prenatal Exposure to Hydroxy-Polycyclic Aromatic Hydrocarbons. Environment International. 2023:107763.

7.	Eaves LA, Bulka CM, Rager JE, Gardner AJ, Galusha AL, Parsons PJ, et al. Metal mixtures modeling identifies birth weight-associated gene networks in the placentas of children born extremely preterm. Chemosphere. 2023;313:137469.

**2^nd^ round**

**Reviewer 1**

In the revised manuscript of "RNAseqCovarImpute: a multiple imputation procedure that outperforms complete case and single imputation differential expression analysis", the authors made considerable efforts to address the reviewers' comments. However, the revised manuscript became a substantially different method from the original, including the following changes:
(1) Synthetic RNA-seq datasets were removed.
(2) Principal components were used instead of binning by genes.

Both of these changes raise many new issues, so I believe that addressing these new issues is necessary to maintain the integrity of the revised manuscript. Here are the main suggestions/questions.

1. The main problem with the current evaluation is that there is no real "real data" analysis. The assumption in the analysis is that there are no systematic confounding effects in the missingness patterns simulated at random or modeled by known covariates. It is still unclear how the method will behave in real data sets. I propose to perform additional experiments by masking a subset of covariates with high missingness and compare the results with the subset of covariates (using the conventional method) and compare the results with the proposed methods when using the full set of covariates (with MI).

2. I am disappointed that the authors have completely removed the synthetic data analysis from the manuscript. This makes it more difficult to understand how the proposed methods work in different scenarios and limits the interpretation of the performance to specific data sets. The generalizability of the method to different types of datasets remains questionable.

3. The effect of selecting the number of PCs is not clearly understood because only two methods were evaluated. Can the authors perform additional experiments by changing the number of PCs selected and evaluate the effect on accuracy? If the authors show that the method is robust to the number of PCs selected (as long as the range is reasonable), this would help readers understand how to select their PCs.

4. Principal components of expression data can be driven by many different factors, such as quality of sample preparation, cell type composition, batch effects, disease status, genetic ancestry, and so on. Not all of these would necessarily be associated with the missing covariates and may introduce new systematic confounding effects when performing differential expression. Is there any reason to believe that the PCA-based method would work better than binning methods in all the different scenarios of expression heterogeneity? Also, would other methods such as PEER be meaningfully more useful?

5. There is no evaluation of the computational efficiency of the proposed methods compared to other standard methods. Computational efficiency is important and needs to be quantified.

**Reviewer 2**

I would like to commend the authors for their exceptional efforts in addressing the reviewers' comments and significantly improving the manuscript. The readability and rigor of the paper have been greatly enhanced. I particularly appreciate the introduction of mathematical formulas to describe the RNAseqCovarImpute process. The authors have also proposed an innovative solution for applying multiple imputation (MI) to high-dimensional gene expression data. The inclusion of two new datasets and the use of robust evaluation metrics in the simulation experiments demonstrate that RNAseqCovarImpute outperforms complete case and single imputation analyses. It effectively uncovers true positive differentially expressed genes, limits false discovery rates, and minimizes bias. Additionally, the well-documented tutorials and the provided R package make the method highly accessible. Overall, I am enthusiastic about this paper and its contributions to the field.

While the authors offer a robust method for predicting missing covariates across multiple simulation scenarios, I still have a few minor suggestions:
1. In the simulation study, I suggest adding a paragraph around line 143 to summarize the advantages of RNAseqCovarImpute in the simulated scenarios.
2. Although the authors have included two RNA-seq datasets in the simulation experiment, it is essential for a bioinformatics tool to demonstrate its generalizability across diverse real-world scenarios. This should include various tissue RNA-seq datasets with multiple covariates, such as cancer or cerebral disease data, in addition to the spontaneous preterm birth dataset.

**Authors’ response**

Reviewer #1

In the revised manuscript of "RNAseqCovarImpute: a multiple imputation procedure that outperforms complete case and single imputation differential expression analysis", the authors made considerable efforts to address the reviewers' comments. However, the revised manuscript became a substantially different method from the original, including the following changes:
(1) Synthetic RNA-seq datasets were removed.
(2) Principal components were used instead of binning by genes.

Thank you for noting this. Yes, the excellent reviewer comments led us down a different but what we believe to be better path for the approach to multiple imputation.

Both of these changes raise many new issues, so I believe that addressing these new issues is necessary to maintain the integrity of the revised manuscript. Here are the main suggestions/questions.

***REVIEWER 1 COMMENT 1***

***A. Reviewer comment***
1. The main problem with the current evaluation is that there is no real "real data" analysis. The assumption in the analysis is that there are no systematic confounding effects in the missingness patterns simulated at random or modeled by known covariates. It is still unclear how the method will behave in real data sets. I propose to perform additional experiments by masking a subset of covariates with high missingness and compare the results with the subset of covariates (using the conventional method) and compare the results with the proposed methods when using the full set of covariates (with MI).

***B. Response***

We agree with the review that it is essential to understand how this method operates in real datasets. We interpret this reviewer comment as a request to evaluate the proposed RNAseqCovarImpute MI method using the full set of covariates against a conventional multivariable model that only adjusts for covariates that have complete data.

In the full model in the real-world example using the ECHO-PATHWAYS data (the section starting at line 205), we adjusted for race, ethnicity, maternal education, family income, tobacco and alcohol use during pregnancy, delivery method, study site, fetal sex, and sequencing batch. To retain all N=1045 in the full data requires a reduced model only adjusting for delivery method, study site, fetal sex, and sequencing batch. This model revealed a substantially different number of DEGs. The original CC, SI, and MI identified 214-575 significant genes associated with maternal age, while this reduced model identified 1071 genes significantly associated with maternal age. We believe that these differences are likely due to confounding by race, ethnicity, and socioeconomic status variables that were not controlled in this analysis.

We also tested dropping the family income variable, which had the most missing data. In the results section we noted that missingness was “mostly driven by 4% (41) of individuals missing family income data.”

The original complete case analysis (including all covariates) identified 362/399 (91%) of the same genes identified in the MI analysis as significant. This new complete case analysis without the income variable, which includes a larger sample size more representative of the sample size from the MI analysis but may be confounded by family income, identified just 270/399 (68%) of the same genes identified in the MI analysis as significant. It also appeared to deviate the most among the methods in terms of ranking the top genes by their p values (the “Drop Income” column in the table below).

In sum, if covariates have high missingness, complete case analysis will result in dropping participants and thus a lower sample size, which could limit power. Alternatively, restricting model coefficients may introduce bias due to uncontrolled variables. We believe these analyses have shown that dropping participants for complete case analysis or imputing missing data is justified to limit bias.

***C. Changes to the text***

We have updated the text to include the model only adjusting for covariates with complete data as:

**Methods** (lines 583-585): “DEGs associated with maternal age while adjusting for all 10 covariates were compared between the CC, SI, and RNAseqCovarImpute MI PCA methods. Additionally, to retain the entire sample size without covariate imputation, a reduced model was fit by omitting any covariates with missing data.”

**Results** (lines 213-220): “Among 1,045 individuals included in this analysis, 6% (61) were missing data for at least one of the 10 covariates, mostly driven by 4% (41) of individuals missing family income data. There were no missing data for delivery method, study site, fetal sex, and sequencing batch, and a minimally adjusted analysis including these variables identified 1,071 DEGs significantly associated with maternal age. Adjusting for all covariates resulted in fewer maternal age DEGs: in the CC, SI, and RNAseqCovarImpute MI analyses, maternal age was associated with 575, 214, and 399 DEGs, respectively (Figure 6A).”

**Discussion** (lines 279-297): “Nevertheless, any of these missing data methods would be a better alternative than omitting covariate control entirely, a common albeit unsatisfactory approach in observational transcriptomic studies. Another option is to only include covariates with complete data, which preserves sample size and avoids the need for imputation but may introduce bias due to uncontrolled variables. For the ECHO-PATHWAYS dataset, only including covariates with complete data resulted in a minimal model adjusting for delivery method, study site, fetal sex, and sequencing batch. This reduced model identified a much larger 1,071 DEGs significantly associated with maternal age compared with 214-575 DEGs in the CC, SI, and RNAseqCovarImpute MI analyses adjusting for all covariates. This larger number of DEGs was likely due to confounding by race, ethnicity, lifestyle, and socioeconomic status variables that were not controlled in this analysis.  Failure to control for these variables in analyses of maternal age could lead to erroneous conclusions and even faulty clinical recommendations. For instance, studies have shown that the positive associations of young maternal age with child ADHD in unadjusted analyses are eliminated or even reversed following adjustment for confounding and mediating variables (28-30). Thus, younger pregnancies do not confer increased ADHD risk because of the biology of aging per se but owing to other variables that are correlated with maternal age. Younger mothers are more likely to smoke during pregnancy, and prenatal tobacco exposure may impair neurodevelopment. If the link between younger pregnancy and adverse child development is mediated by increased tobacco exposure, then clinical efforts focusing on reducing tobacco exposures during pregnancy would be more effective than recommendations regarding the ideal age for childbearing (28).”

***REVIEWER 1 COMMENT 1 (continued)***

***A. Reviewer comment***

Regarding the concern that there is no real "real data" analysis:

***B. Response***

We apologize that we didn’t make clear that real world data is used in the results section. There are 3 real data analyses, followed by simulations to induce missingness in the data, and then the application of the various methods to deal with missing data tested here (MI, SI, and complete case). We describe the analysis of the real data in the methods lines 462-474).

***C. Changes to the text***

To make the real data analyses clearer, we have changed the name of the “Simulation study” results section to “Performance on three example datasets”. We have also added some details of these analyses to the beginning of this section in the results (lines 102-129):

“**Performance on three example datasets**

Three large real-world RNA-sequencing datasets encompassing multiple tissue types and a diverse range of covariates were utilized to compare RNAseqCovarImpute, SI, and CC differential expression analysis (Methods).

*ECHO PATHWAYS Dataset:* The ECHO prenatal and early childhood pathways to health (ECHO-PATHWAYS) dataset (dbGaP phs003619.v1.p1 and phs003620.v1.p1) includes RNA-sequencing of placentas sampled at delivery from socioeconomically and racially/ethnically diverse participants from two regionally distinct birth cohorts in Washington (Seattle and Yakima) and Tennessee (Memphis), USA (5). For the ECHO-PATHWAYS dataset (N=994), maternal age served as the predictor of interest in differential expression analysis, while covariates included fetal sex, RNA-sequencing batch, maternal tobacco use during pregnancy, maternal alcohol use during pregnancy, and family income.

*NSCLC Dataset:* The non-small cell lung cancer (NSCLC) dataset (EMBL-EBI: E-GEOD-81089) includes RNA-sequencing of both lung tumor and non-malignant tissues sampled from patients diagnosed with NSCLC being surgically treated from 2006 to 2010 at the Uppsala University Hospital, Sweden (16). For the NSCLC dataset (N=670), the predictor of interest was sex, while covariates included participant age, participant smoking status, and sampling site (tumor versus non-malignant).

*EBV Dataset:* The Epstein-Barr virus (EBV) dataset (EMBL-EBI: E-MTAB-7805) analyzed primary cultures of human B lymphocytes obtained from adenoid tissue (17). For the EBV dataset (N=384), the predictor of interest was time elapsed in culture, while covariates included EBV infection status and individual donor source.

The ECHO-PATHWAYS dataset included 14,026 genes after filtering, of which 2,517 were significantly associated with maternal age in the full data model (true positives) while adjusting for covariates. Following the full data model, simulations to induce missingness in the covariate data were performed 10 times per level of missing data and missingness mechanism (Methods).”

***REVIEWER 1 COMMENT 1 (continued)***

***A. Reviewer comment***

Regarding the concern that the assumption in the analysis is that there are no systematic confounding effects in the missingness patterns simulated at random or modeled by known covariates

***B. Response***

The real data analyses utilized in the simulation study (described above) are subject to confounding by uncontrolled factors. For example, the ECHO PATHWAYS dataset analysis in the simulation study included fetal sex, RNA-sequencing batch, maternal tobacco use during pregnancy, maternal alcohol use during pregnancy, and family income as covariates. However, we included an expanded set of covariates in the “Application of RNAseqCovarImpute in analysis of maternal age and placental transcriptome” section starting on line 205 of the results.

While the simulation study results are technically subject to bias, including from confounding, we take the result as the ground truth in this case because the goal of imputing missing data here is to best replicate the results we would have obtained if there were no missing data in the first place.

Additionally, the simulations where missing data were induced under MAR and MNAR mechanisms do in fact have systematic confounding effects in the missingness patterns. We give an example of this in the text (lines 130-134):

“When data were simulated to be missing at random (MAR) or missing not at random (MNAR), the maternal alcohol and family income variables had strong influence over patterns of missing data as intended. For example, in the simulated datasets where 55% of individuals had at least one missing data point, the average rate of individuals with at least one missing data point was 91% among alcohol users but only 50% among those reporting no alcohol use.”

***REVIEWER 1 COMMENT 2***

***A. Reviewer comment***

2. I am disappointed that the authors have completely removed the synthetic data analysis from the manuscript. This makes it more difficult to understand how the proposed methods work in different scenarios and limits the interpretation of the performance to specific data sets. The generalizability of the method to different types of datasets remains questionable.

***B. Response***

We agree that analyzing more data with different properties can better probe the generalizability of the method.

***C. Changes to the text***

In response to this comment, we have updated the manuscript to include synthetic RNA-sequencing data that are described in the methods as (lines 560-569):

“Finally, four sets of synthetic RNA-sequencing data were also used to compare performances of RNAseqCovarImpute (MI PCA Horn method), SI, and CC differential expression analysis. The NSCLC RNA-sequencing data were modified to add known signal using the seqgendiff package (18). The method relies on binomial thinning of the RNA-sequencing count matrix to closely match user defined coefficients. Rather than generating counts from theoretical distributions, thinning a real set of RNA-sequencing counts can better preserve realistic variability and inter-gene correlations typical of RNA-sequencing data (18). Subsets of 25%, 50%, 75%, or 99% of genes were randomly selected to have their coefficient of association (Log2 fold-changes) with sex set to zero. The remaining coefficients were drawn randomly from a gamma distribution generated using rgamma(shape = 1) from the stats package in R.”

In supplemental figures 5 and 6 we plot summary/diagnostic information for these synthetic data, while supplemental figures 7 and 8 show that the new MI method has generally higher true positive rates and lower false positive rates compared with CC and SI. This is described in the results as (lines 176-189)

“Distributions of gene expression coefficients associated with sex depended on the desired null gene rates for each synthetic dataset, but followed a similar form compared with the original NSCLC data as intended (Supplemental Figure 5). Coefficients for the remaining genes were drawn randomly from a gamma distribution, and an additional diagnostic confirmed that the coefficients for each gene estimated from the limma-voom pipeline on the synthetic count tables closely matched these pre-defined coefficients input into the seqgendiff package (Supplemental Figure 6). Applied to these synthetic RNA-sequencing datasets, RNAseqCovarImpute had higher TPRs (Supplemental Figure 7) and lower FPRs (Supplemental Figure 8) compared to CC and SI differential expression analysis across most scenarios. Moreover, the advantages of RNAseqCovarImpute were most apparent for synthetic datasets with weaker signals. For example, all methods had FPRs <0.05 for the synthetic data with the strongest signal (i.e., 75% of genes associated with the predictor of interest and only 25% null genes). However, with 99% null genes and only 1% of genes simulated to correlate with the predictor of interest, RNAseqCovarImpute maintained FPRs <0.05 while the other methods did not (Supplemental Figure 8).”

***REVIEWER 1 COMMENT 3***

***A. Reviewer comment***
3. The effect of selecting the number of PCs is not clearly understood because only two methods were evaluated. Can the authors perform additional experiments by changing the number of PCs selected and evaluate the effect on accuracy? If the authors show that the method is robust to the number of PCs selected (as long as the range is reasonable), this would help readers understand how to select their PCs.

***B. Response***

We agree that we should provide more information on selecting the number of PCs to retain, as this could vary by dataset and there are many methods that researchers use to make this decision. We have updated the supplemental results to present three different methods for determining the number of PCs to retain: Horn’s parallel analysis, 80% variance explained cutoff, and the elbow method. Interestingly, we saw great stability in the results even with substantial differences in the number of PCs retained, at least at low levels of missing data.

***C. Changes to the text***

Methods (Lines 371-375):

“Performance of MI PCA was compared when using Horn’s parallel analysis, an 80% variance explained cutoff, and the elbow method, where all PCs are retained that come before the elbow point in the curve of variance explained by each successive PC. Methods for determining the number of retained PCs were implemented using Bioconductor’s PCAtools R package (13).”

Results lines 89-101

“Three versions of MI PCA using different criteria to determine the number of retained PCs were compared with the MI Gene Bin approach (Methods). MI PCA using Horn’s parallel analysis performed better than the MI Gene Bin and other MI PCA methods. All methods had similar TPRs, while MI PCA horn had the lowest FPRs across most scenarios (Supplemental Results, Supplemental Figures 2-4). Among the methods for retaining PCs, results were relatively comparable when missing data were minimal. For instance, for the ECHO-PATHWAYS dataset, Horn’s parallel analysis retained 35 PCs, an 80% variance explained cutoff retained 213 PCs, and the elbow method retained 15 PCs. Despite substantial differences in the number of retained PCs, all methods had similarly high TPRs and good FPR control at approximately 0.05 when there was only 5-15% missing data (Supplemental Figure 2). However, when levels of missing data were higher, FPRs were consistently controlled at 0.05 for Horn’s parallel analysis, but not for the 80% variance explained or elbow approaches (Supplemental Figure 2). Thus, MI PCA using Horn’s parallel analysis was selected as the MI method of choice (hereinafter the “RNAseqCovarImpute” method).”

We also updated the limitations paragraph of the discussion to mention this (lines 306-311):

“One limitation to RNAseqCovarImpute is that it is dependent on selection of a number of PCs to integrate, and the user will need to define the optimal number of PCs to retain using established methods such as Horn’s parallel analysis or a variance explained cutoff. Our testing suggests that several popular methods perform well when missing data is minimal. At high levels of missing data, Horn’s parallel analysis was the best general method of choice, but performance could vary with different datasets.”

***REVIEWER 1 COMMENT 4***

***A. Reviewer comment***
4. Principal components of expression data can be driven by many different factors, such as quality of sample preparation, cell type composition, batch effects, disease status, genetic ancestry, and so on. Not all of these would necessarily be associated with the missing covariates and may introduce new systematic confounding effects when performing differential expression. Is there any reason to believe that the PCA-based method would work better than binning methods in all the different scenarios of expression heterogeneity? Also, would other methods such as PEER be meaningfully more useful?

***B. Response***

Our results have demonstrated that the gene binning method would be subject to many of these same issues related to sample preparation, batches, etc., as the gene expression data are still used in the multiple imputation predictive models, just in another format. Additionally, in multiple imputation, including auxiliary variables—those not directly related to the main outcome or predictors—can enhance the accuracy of the imputation process by providing additional information that helps predict missing values more reliably. The auxiliary variables can account for relationships and dependencies within the data that might otherwise be overlooked (Graham, 2009).

PEER is an interesting idea to consider, although we have thought about the fact that the goal of PCA here is to include information on the outcome data in the imputation predictive modeling, not for confounder adjustment. Having the outcome included in the imputation predictive model is a requirement of multiple imputation. As we understand, PEER is designed to uncover and control for unobserved confounders, inferred from the gene expression data. In our situation, it is the gene expression data itself that is of interest to include in the MI predictive models, and PCA seemed a better choice because it is meant to explain maximal variance. PEER factors could still be entered into the RNAseqCovarImpute analysis as confounders, along with the other clinical/demographic covariates.

***REVIEWER 1 COMMENT 5***

***A. Reviewer comment***
5. There is no evaluation of the computational efficiency of the proposed methods compared to other standard methods. Computational efficiency is important and needs to be quantified.

***B. Response***

Thank you for pointing this out, we have added a “Computational benchmarks” section.

***C. Changes to the text***

Results (lines 197-203)

“**Computational benchmarks**

Methods were benchmarked in an analysis of the ECHO-PATHWAYS dataset with 14,026 genes, 994 observations, 4 covariates in the model, and 55% missingness under MCAR on a Windows machine with 3.8 GHz processing speed and 16GB random-access memory. Over three iterations per method, memory allocations and median run times were 16.6GB and 13.52 minutes for the RNAseqCovarImpute MI Gene Bin method, 4.32GB and 2.68 minutes for the RNAseqCovarImpute MI PCA method, 7.58GB and 17.46 seconds for SI, and 3.23GB and 6.71 seconds for CC.”

We have also added a short sentence on this to the limitations section of the discussion (Lines 323-324):

“Another limitation was that, as an MI method, RNAseqCovarImpute required more processing time compared with CC or SI.”

Reviewer #2

I would like to commend the authors for their exceptional efforts in addressing the reviewers' comments and significantly improving the manuscript. The readability and rigor of the paper have been greatly enhanced. I particularly appreciate the introduction of mathematical formulas to describe the RNAseqCovarImpute process. The authors have also proposed an innovative solution for applying multiple imputation (MI) to high-dimensional gene expression data. The inclusion of two new datasets and the use of robust evaluation metrics in the simulation experiments demonstrate that RNAseqCovarImpute outperforms complete case and single imputation analyses. It effectively uncovers true positive differentially expressed genes, limits false discovery rates, and minimizes bias. Additionally, the well-documented tutorials and the provided R package make the method highly accessible. Overall, I am enthusiastic about this paper and its contributions to the field.

Thank you, we agree that the manuscript has been improved following the helpful guidance from excellent reviewer comments.

While the authors offer a robust method for predicting missing covariates across multiple simulation scenarios, I still have a few minor suggestions:

***REVIEWER 2 COMMENT 1***

***A. Reviewer comment***

1.      In the simulation study, I suggest adding a paragraph around line 143 to summarize the advantages of RNAseqCovarImpute in the simulated scenarios.

***B. Response***

Thanks for the suggestion, we see how the main message could be lost within descriptions of the results from different datasets.

***C. Changes to the text***

We have added the following to the end of the simulation study section of the results (lines 191-195):

“Overall, RNAseqCovarImpute outperformed SI and CC methods in differential expression analysis by achieving higher TPRs, lower FPRs, and lower MAPEs across various real-world and synthetic RNA-sequencing datasets. Its advantages were most notable, especially with respect to controlling FPRs, in scenarios with high levels of missing data or predictors of interest that are only weakly associated with the RNA-sequencing data.”

***REVIEWER 2 COMMENT 2***

***A. Reviewer comment***
2.      Although the authors have included two RNA-seq datasets in the simulation experiment, it is essential for a bioinformatics tool to demonstrate its generalizability across diverse real-world scenarios. This should include various tissue RNA-seq datasets with multiple covariates, such as cancer or cerebral disease data, in addition to the spontaneous preterm birth dataset.

***B. Response***

We agree with this reviewer comment, and our method is relevant across diverse real world scenarios as we describe. We have added text to the results section to describe the three datasets utilized here in more depth and to emphasize the diversity of sample types and study designs, which includes placental tissues, lung cancer tissue, non-malignant biopsies from individuals with lung cancer, and primary cell cultures.

***C. Changes to the text***

Results (lines 102-129):

“**Performance on three example datasets**

Three large real-world RNA-sequencing datasets encompassing multiple tissue types and a diverse range of covariates were utilized to compare RNAseqCovarImpute, SI, and CC differential expression analysis (Methods).

*ECHO PATHWAYS Dataset:* The ECHO prenatal and early childhood pathways to health (ECHO-PATHWAYS) dataset (dbGaP phs003619.v1.p1 and phs003620.v1.p1) includes RNA-sequencing of placentas sampled at delivery from socioeconomically and racially/ethnically diverse participants from two regionally distinct birth cohorts in Washington (Seattle and Yakima) and Tennessee (Memphis), USA (5). For the ECHO-PATHWAYS dataset (N=994), maternal age served as the predictor of interest in differential expression analysis, while covariates included fetal sex, RNA-sequencing batch, maternal tobacco use during pregnancy, maternal alcohol use during pregnancy, and family income.

*NSCLC Dataset:* The non-small cell lung cancer (NSCLC) dataset (EMBL-EBI: E-GEOD-81089) includes RNA-sequencing of both lung tumor and non-malignant tissues sampled from patients diagnosed with NSCLC being surgically treated from 2006 to 2010 at the Uppsala University Hospital, Sweden (16). For the NSCLC dataset (N=670), the predictor of interest was sex, while covariates included participant age, participant smoking status, and sampling site (tumor versus non-malignant).

*EBV Dataset:* The Epstein-Barr virus (EBV) dataset (EMBL-EBI: E-MTAB-7805) analyzed primary cultures of human B lymphocytes obtained from adenoid tissue (17). For the EBV dataset (N=384), the predictor of interest was time elapsed in culture, while covariates included EBV infection status and individual donor source.

The ECHO-PATHWAYS dataset included 14,026 genes after filtering, of which 2,517 were significantly associated with maternal age in the full data model (true positives) while adjusting for covariates. Following the full data model, simulations to induce missingness in the covariate data were performed 10 times per level of missing data and missingness mechanism (Methods).”

Reference:

Graham, John W. "Missing data analysis: Making it work in the real world." *Annual review of psychology* 60 (2009): 549-576.

**3^rd^ round**

**Reviewer 1**

The authors have made commendable efforts to address each point in the revised manuscript. I have a few minor suggestions for the final version, which the authors and editors may consider at their discretion:

1. The "Drop Income" analysis in the response to my "real data analysis" request is most valuable and merits inclusion in the final paper. Dropping all covariates even with few missing values may not be practical, and some might argue that dropping income is a best practice for the ECHO-PATHWAY dataset when imputation is not feasible.

2. Regarding the ECHO-PATHWAY analysis in Fig. 6, it would be beneficial to provide a clearer explanation of why the "Complete Case" experiment results in more differentially expressed genes than other methods, and why these genes are considered less credible than those identified through Multiple Imputation (MI) or Single Imputation (SI). There is an apparent paradox that including more samples with MI doesn't necessarily increase power compared to CC as expected. Consider discussing the possibility of overcorrection in the proposed method, as the disappearance of some differentially expressed genes after MI could suggest this. A more nuanced interpretation would help readers understand that RNAseqCovar's MI may not always be more powerful than Complete Case analysis in the context where overcorrection may happen.

3. The evaluation of computational efficiency is helpful and indicates that computational cost is not a major bottleneck for the given sample size. To encourage application to larger datasets, consider providing:
a) A theoretical time complexity formula (e.g., O(n^2 mr), where n is sample size, m is number of genes, and r is the number of multiple imputations), and/or
b) An empirical graph of computational cost based on available observations.
This information would help users understand any quadratic or cubic complexities involved in certain steps.

4. Consider commenting on potential extensions of RNAseqCovar to multi-omics or other high-dimensional data settings. Discussing additional considerations for such expansions could increase interest in this valuable tool.

**Reviewer 2**

I would like to commend the authors for their outstanding efforts in addressing the reviewers' comments and significantly improving the manuscript. However, I still have a few commnents:

(1) It is confusing that the authors changed the title "Simulation study" to "Performance on three example datasets" on line 102. In this section, the authors designed several missing patterns of covariates in three real RNA-seq datasets and evaluated the performance of RNAseqCovarImpute. Instead of real missing covariates shown in the datasets, the missing elements were artificially designed. Therefore, I suggest that the authors carefully design the title of this section to clearly distinguish between the simulation study and the real data study.

(2) As a bioinformatics tool for the imputation of covariates in RNA-sequencing data, it is crucial for the authors to verify the generalizability of their method in real-world situations. Currently, the authors have only applied RNAseqCovarImpute to the ECHO PATHWAYS dataset for the analysis of maternal age and the placental transcriptome. I strongly recommend that the authors include new sections exploring cancer or brain diseases and identify meaningful DEGs associated with these diseases in real-world scenarios, instead of focusing solely on simulation studies. In the real world, if a method can only be applied to the analysis of a single dataset (ECHO PATHWAYS dataset), its significance will dramatically decrease.

**Authors’ response**

Reviewer #1: The authors have made commendable efforts to address each point in the revised manuscript. I have a few minor suggestions for the final version, which the authors and editors may consider at their discretion:

1. The "Drop Income" analysis in the response to my "real data analysis" request is most valuable and merits inclusion in the final paper. Dropping all covariates even with few missing values may not be practical, and some might argue that dropping income is a best practice for the ECHO-PATHWAY dataset when imputation is not feasible.

We have updated the manuscript to include the analyses where income was dropped as a covariate. The P-value rankings and volcano plot for this analysis are reported alongside the other methods in Figure 6. We also added Figure S10, a 4-way Venn diagram depicting overlapping DEGs between the CC, SI, MI, and “Drop Income” analyses. The Drop Income analysis is described in the methods:

“Additionally, an alternative CC analysis was performed while omitting family income, the variable with the most missing data.”

In the results:

“Additionally, CC analysis was repeated while omitting the family income covariate, which preserved sample size while allowing possible confounding by this variable. This analysis uncovered 334 DEGs, of which 68% (270) overlapped with the DEGs from the MI method (Figure S10). Although there were some differences, genes ranked from lowest to highest P-value followed similar orders between the methods (Figure 6B). The most substantial differences compared with the P-value rank order from the MI analysis were observed in the fully adjusted CC and CC omitting family income analyses (Figure 6B).”

In the discussion:

“Nevertheless, any of these missing data methods would be a better alternative than omitting covariate control entirely, a common albeit unsatisfactory approach in observational transcriptomic studies. Another reasonable alternative is to perform CC analysis while omitting variables with the most missing data. Compared with fully adjusted CC analysis, CC while omitting the family income variable was more similar to the MI analysis in terms of the total number of DEGs, but displayed more differences in terms of the P-value rank order of DEGs.”

2. Regarding the ECHO-PATHWAY analysis in Fig. 6, it would be beneficial to provide a clearer explanation of why the "Complete Case" experiment results in more differentially expressed genes than other methods, and why these genes are considered less credible than those identified through Multiple Imputation (MI) or Single Imputation (SI). There is an apparent paradox that including more samples with MI doesn't necessarily increase power compared to CC as expected. Consider discussing the possibility of overcorrection in the proposed method, as the disappearance of some differentially expressed genes after MI could suggest this. A more nuanced interpretation would help readers understand that RNAseqCovar's MI may not always be more powerful than Complete Case analysis in the context where overcorrection may happen.

We have updated the discussion to consider higher power of CC as a possibility, but to explain why we believe that the higher number of DEGs could also be explained by higher false positive rates in CC analysis, which was supported in the simulations.

Discussion:

“We applied RNAseqCovarImpute to a large observational study of maternal age and the placental transcriptome. This analysis assessed the association of maternal age with placental gene expression controlling for confounding variables such as maternal race, ethnicity, and socioeconomic status, and potential mediators such as alcohol and tobacco use during pregnancy. Although there was some overlap, the MI analysis uncovered a different set of differentially expressed genes compared with the CC and SI analyses. CC analysis uncovered a larger number of DEGs compared with MI, possibly indicating that CC may have higher power in some cases. However, the simulations suggest that CC analyses have higher FPRs across many scenarios compared with MI via RNAseqCovarImpute. Individuals with missing data could systematically differ from those with complete data, and dropping these individuals could result in bias. Although higher power of CC remains a possibility, it is more likely that the excess number of DEGs in the CC analysis could be explained by false- positives owing to such bias. Ultimately, in a real-world example, the method for dealing with missing data matters, and our simulations suggest that MI should be the preferred approach.”

3. The evaluation of computational efficiency is helpful and indicates that computational cost is not a major bottleneck for the given sample size. To encourage application to larger datasets, consider providing:

a) A theoretical time complexity formula (e.g., O(n^2 mr), where n is sample size, m is number of genes, and r is the number of multiple imputations), and/or

b) An empirical graph of computational cost based on available observations. This information would help users understand any quadratic or cubic complexities involved in certain steps.

We have added a supplemental figure depicting the computation time of the RNAseqCovarImpute MI PCA method while varying the sample size (N = 300, 600 or 994), the number of genes (4675, 9350, or 14026 genes), and the number of imputations (M = 10, 20, 30, or 100). This is now described in the results as:

“Computation time was further assessed for the RNAseqCovarImpute MI PCA method over several combinations of sample size, number of genes, and number of imputed datasets (Figure S9).”

4. Consider commenting on potential extensions of RNAseqCovar to multi-omics or other high-dimensional data settings. Discussing additional considerations for such expansions could increase interest in this valuable tool.

We have added the following sentence to the end of the conclusions section:

“Future studies may expand upon the MI methods developed here for RNA-sequencing to address problems associated with missing covariate data in other settings, including DNA methylation, proteomics, metabolomics, and other high dimensional data types.”

Reviewer #2: I would like to commend the authors for their outstanding efforts in addressing the reviewers' comments and significantly improving the manuscript.

However, I still have a few commnents:

(1) It is confusing that the authors changed the title "Simulation study" to "Performance on three example datasets" on line 102. In this section, the authors designed several missing patterns of covariates in three real RNA-seq datasets and evaluated the performance of RNAseqCovarImpute. Instead of real missing covariates shown in the datasets, the missing elements were artificially designed. Therefore, I suggest that the authors carefully design the title of this section to clearly distinguish between the simulation study and the real data study.

We have renamed this section to be more descriptive as:

“Performance on three real datasets following simulations of missing covariate data”

(2) As a bioinformatics tool for the imputation of covariates in RNA-sequencing data, it is crucial for the authors to verify the generalizability of their method in real-world situations. Currently, the authors have only applied RNAseqCovarImpute to the ECHO PATHWAYS dataset for the analysis of maternal age and the placental transcriptome. I strongly recommend that the authors include new sections exploring cancer or brain diseases and identify meaningful DEGs associated with these diseases in real-world scenarios, instead of focusing solely on simulation studies. In the real world, if a method can only be applied to the analysis of a single dataset (ECHO PATHWAYS dataset), its significance will dramatically decrease.

In response to previous suggestions by reviewers that encouraged other examples using real world data, we have added another real-world example analysis based on data downloaded from the European Molecular Biology Laboratory - European Bioinformatics Institute (EMBL-EBI). These data come from an RNA-sequencing study of blood platelets in individuals with cancer and healthy donors. The dataset contains real missing values for the sex and age covariates.

Methods:

“Application of RNAseqCovarImpute in analysis of colorectal carcinoma and the blood platelet transcriptome In another real-world example, RNAseqCovarImpute was applied to a dataset of blood platelet RNA-sequencing from 42 individuals with colorectal carcinoma and 59 healthy donors (EMBL-EBI: E-GEOD-68086) (20). This analysis examined the association of colorectal carcinoma versus healthy cancer status with the transcriptome while controlling for genotype (KRAS vs. PIK3CA vs. wild-type), sex and age (N=101). DEGs associated with colorectal carcinoma while adjusting for these covariates were compared between the CC, SI, and RNAseqCovarImpute MI PCA methods.”

Results:

“Application of RNAseqCovarImpute in analysis of colorectal carcinoma and the blood platelet transcriptome In another real-world example, RNAseqCovarImpute was applied to a dataset of blood platelet RNA-sequencing from 42 individuals with colorectal carcinoma and 59 healthy donors (20). This dataset included 3,227 genes after filtering. There were 14 individuals with KRAS mutant tumors and 2 individuals with PIK3CA mutant tumors compared with 85 wild-type individuals. No data were missing for cancer status or genotype, while 34% (34) were missing data for sex, and 40% (40) were missing data for age. In the CC, SI, and RNAseqCovarImpute MI analyses, colorectal carcinoma was associated with 2,491, 2,403, and 2,579 DEGs, respectively, while controlling for genotype, sex, and age (Figure S12A). The CC and SI analyses uncovered 94% (2,422) and 92% (2,360) of the significant DEGs from the MI method, respectively, while there were 98 DEGs exclusive to MI (Figure S12A). Genes ranked from lowest to highest P-value followed similar orders between the methods (Figure S12B). Many of the top DEGs in the MI analysis, according to their significance and fold- change magnitude (Figure S12C-E), have been previously shown to play roles in cancer etiology. For example, colorectal carcinoma was associated with the downregulation of GK5. Genes involved in glycerol metabolism, including GK5, have been previously implicated in the etiology of cancers, including colorectal carcinoma (21-24). Mutations in MDN1, which was downregulated in association with colorectal cancer here, have been shown to correlate with elevated tumor mutation rates in breast and colorectal cancers (25, 26). Downregulation of TNFRSF1B (a member of the tumor necrosis factor gene family) in association with colorectal cancer here is consistent with prior studies showing lower mRNA expression of TNFRSF1B in lung cancer compared with normal lung tissue (27).”

Discussion:

“To the best of our knowledge, MI via RNAseqCovarImpute is applicable to any RNA- sequencing study that has missing values in the paired covariate data. In addition to the placental transcriptomics data in ECHO-PATHWAYS, we applied RNAseqCovarImpute to three different datasets. In one example, we analyzed blood platelet RNA-sequencing data from individuals with colorectal carcinoma and healthy controls (20). This analysis uncovered several differentially expressed genes with known roles in the etiology and progression of various cancers, including colorectal carcinoma. Future studies may utilize RNAseqCovarImpute to achieve higher power and lower FPRs in differential gene expression analyses of a wide range of factors and across diverse tissue types.”
